# Chemical synthesis of the pentasaccharide repeating unit of the *O*-specific polysaccharide from *Escherichia coli* O132 in the form of its 2-aminoethyl glycoside

**DOI:** 10.3762/bjoc.15.249

**Published:** 2019-10-28

**Authors:** Debasish Pal, Balaram Mukhopadhyay

**Affiliations:** 1 Sweet Lab, Department of Chemical Sciences, Indian Institute of Science Education and Research (IISER) Kolkata, Mohanpur, Nadia 741246, India

**Keywords:** 2-aminoethyl glycoside, diarrhea, galactofuranose, O-specific polysaccharide, total synthesis

## Abstract

The total chemical synthesis of the pentasaccharide repeating unit of the *O*-polysaccharide from *E. coli* O132 is accomplished in the form of its 2-aminoethyl glycoside. The 2-aminoethyl glycoside is particularly important as it allows further glycoconjugate formation utilizing the terminal amine without affecting the stereochemistry of the reducing end. The target was achieved through a [3 + 2] strategy where the required monosaccharide building blocks are prepared from commercially available sugars through rational protecting group manipulation. The NIS-mediated activation of thioglycosides was used extensively for the glycosylation reactions throughout.

## Introduction

*O*-Polysaccharides are the most complex molecular systems present in the bacterial cell wall owing to the presence of various sugar residues connected through diverse glycosidic linkages. These complex structures play a key role in determining and regulating the biology of these organisms [1] and act as the elicitor of the innate immune response [2]. As these polysaccharides can protect the bacteria concerned by killing the serum complements of the host system and can stop phagocytosis, they are extremely important for the pathogenicity of the microorganisms [2]. Their antigenic character has made them attractive targets for designing potential vaccine candidates. Arguably these complex structures can be isolated from the biological sources, however, often this isolation is cumbersome and becomes too expensive to get reasonable quantities of material with adequate purity. Therefore, the chemical syntheses of these complex structures become the only option to afford the material for detailed biological studies leading to the exploration of the vaccine potential.

**Figure 1:**
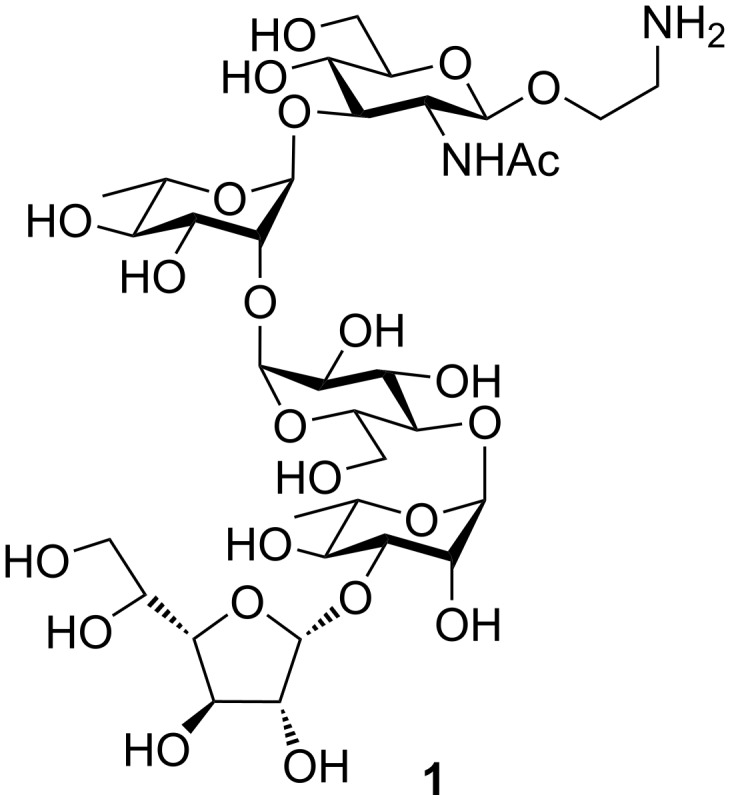
Structure of the target pentasaccharide repeating unit in the form of its 2-aminoethyl glycoside.

*Escherichia coli* is a Gram-negative bacteria present in the guts of human and other warm-blooded animals. It is usually harmless and beneficial to the host’s body, however, there are other variants of *E. coli* having virulence factors and causing diseases like diarrhea, urinary tract infection, septicaemia etc. *E. coli* O132 is reported to be the causative agent for septicemia in chicken [3], diarrhea in children under three years of age [4], diarrhea in calves and rabbits [5–6]. Recently, Knirel et al. [7] reported the structure of the *O*-polysaccharide of *E. coli* O132 as a pentasaccharide repeating unit consisting of GlcNAc*p*, Glc*p*, Rha*p* and Gal*f*. In this communication we report on the total synthesis of this pentasaccharide repeating unit of the *O*-specific polysaccharide in the form of its 2-aminoethyl glycoside (Figure 1) through a [3 + 2] converging strategy. The building blocks were prepared by suitable protecting group manipulations on the commercially available monosaccharides and stereoselective chemical glycosylations. The 2-aminoethyl glycoside at the reducing end will facilitate further glycoconjugate formation without hampering the stereochemistry of the anomeric center. We have used similar glycosides in case of other chemically synthesized oligosaccharides before [8–10]. The corresponding aminopropyl linker has also been used by others [11]. The chemically synthesized oligosaccharide structure will help to elucidate further biological implications of the *O*-antigen concerned and possible vaccine potential.

## Results and Discussion

**Figure 2:**
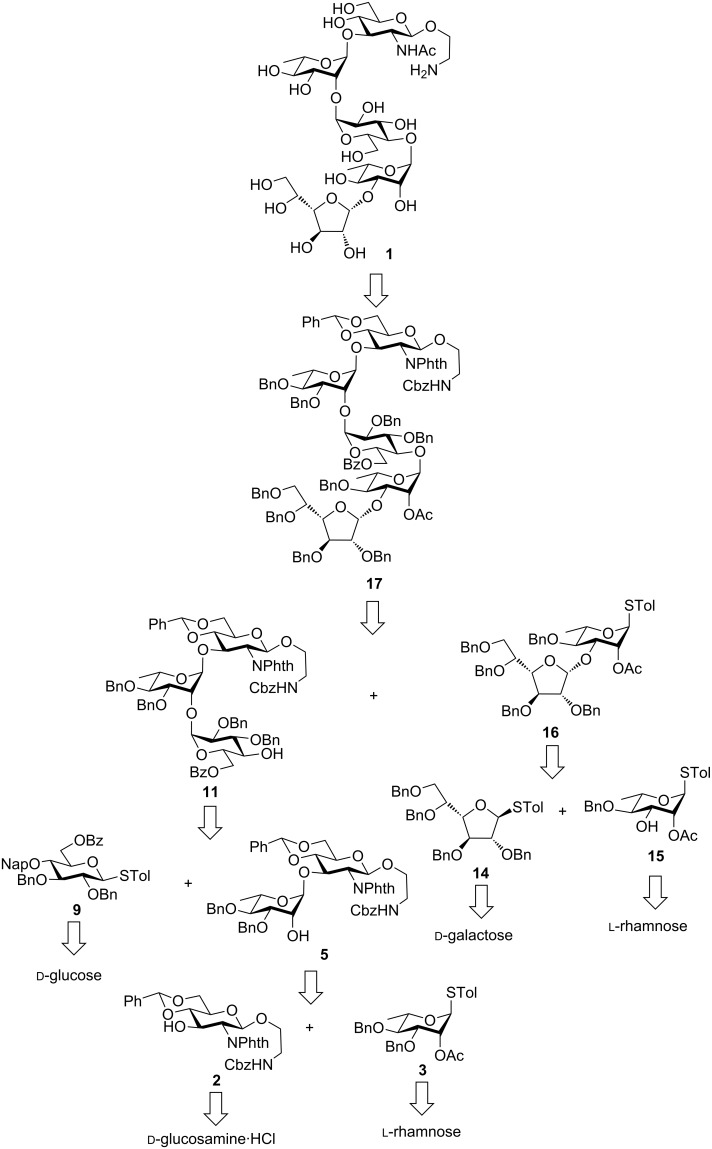
Retrosynthetic analysis for the synthesis of the target pentasaccharide **1**.

It is important to select a suitable glycoside at the reducing end of the target oligosaccharide keeping in mind that the glycoside should allow further glycoconjugate formation without disturbing the stereochemistry of the anomeric center. Therefore, the 2-aminoethyl glycoside was selected at the reducing end of the target pentasaccharide that will allow further conjugation using the terminal amine without affecting the glycosidic stereochemistry. Further retrosynthetic analysis of the target pentasaccharide **1** revealed that a [3 + 2] strategy will be the most suitable one for the total synthesis of the aforementioned target. Therefore, the first disconnection gave the trisaccharide acceptor **11** and the disaccharide donor **16**. Further disconnection on the trisaccharide **11** gave disaccharide **5** and the monosaccharide **9**. Disaccharide **5** is accessible from monosaccharide acceptor **2** and donor **3**. Similarly, the disaccharide donor **16** may be prepared from monosaccharide acceptor **15** and donor **14**. The monosaccharide building blocks can be prepared from commercially available ᴅ-glucose, ᴅ-galactose, ᴅ-glucosamine hydrochloride and ʟ-rhamnose through rational protecting group manipulations (Figure 2).

**Scheme 1:**
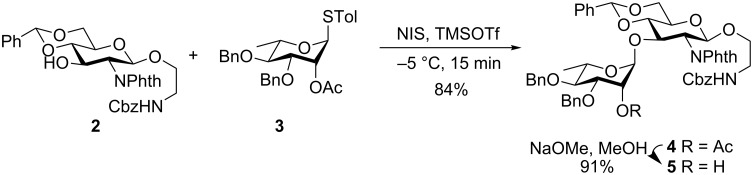
Synthesis of the disaccharide acceptor **5**.

Glycosylation of the known GlcNPhth acceptor **2** [12] and the rhamnosyl donor **3** [13] through the activation of thioglycoside using *N*-iodosuccinimide (NIS) in the presence of TMSOTf gave the Rha-(1→3)-GlcNPhth disaccharide **4** in 84% yield. The presence of a participating acetate group at the 2-position of the rhamnosyl donor **3** ensured the formation of the desired 1,2-*trans* glycoside. Peaks at 4.52 ppm (s, 1H, H-1′) in the ^1^H NMR and at 98.1 (C-1′) in the ^13^C NMR spectra confirmed the stereochemistry of the newly formed glycosidic linkage. Further, Zemplén de-*O*-acetylation [14] gave the disaccharide acceptor **5** in 91% isolated yield (Scheme 1).

**Scheme 2:**
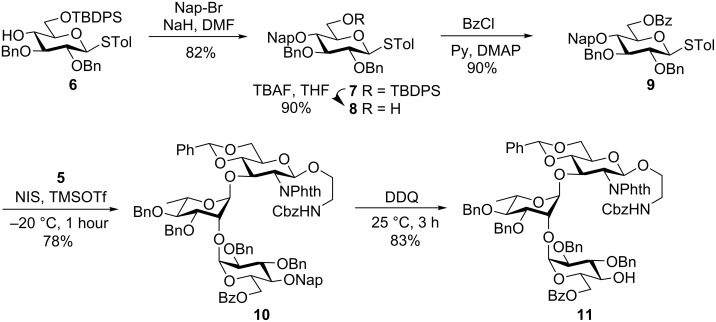
Synthesis of the trisaccharide acceptor **11**.

In a separate experiment, the 4-*O*-hydroxy group of the known compound **6** [15] was alkylated using naphthyl bromide in the presence of NaH [16] to afford the corresponding naphthyl derivative **7** in 82% yield. Next, the TBDPS group was removed using tetrabutylammonium fluoride in THF [17] to give the 6-hydroxy compound **8** in 90% yield. It was then benzoylated using BzCl in pyridine [18] with a catalytic amount of DMAP to furnish the completely protected donor **9** in 90% yield. Glycosylation of donor **9** with disaccharide acceptor **5** through activation of the thioglycoside using NIS and TMSOTf afforded the trisaccharide **10** in 78% yield. The newly formed 1,2-*cis* glycosidic linkage was confirmed by the peaks at 4.27 ppm (d, *J*_1′′,2′′_ = 1.5 Hz, 1H, H-1′′) in the ^1^H NMR and at 93.8 ppm (C-1′′) in the ^13^C NMR spectra. The presence of the participating *O*-benzoyl group at the 6-*O*-position of the donor led exclusively to the 1,2-*cis* glycoside [19]. Finally, the oxidative removal of the naphthyl group using DDQ [20] afforded the trisaccharide acceptor **11** in 83% yield (Scheme 2).

**Scheme 3:**
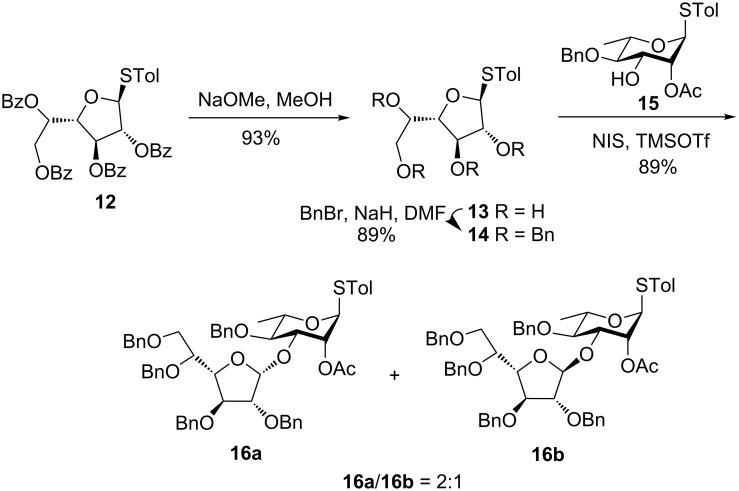
Synthesis of the non-reducing end disaccharides (**16a** and **16b**).

The known galactofuranosyl derivative **12** [21] was prepared by following a literature procedure. It was subjected to Zemplén de-*O*-benzoylation using NaOMe in MeOH to give compound **13** in 93% yield. Further, per-*O*-benzylation using BnBr in the presence of NaH [22] afforded the galactofuranosyl donor **14** in 89% yield. Glycosylation of the donor **14** with known rhamnose acceptor **15** [23] through activation of the thioglycoside using NIS in the presence of TMSOTf at low temperature gave the disaccharide **16a** (α) [^1^H NMR: 5.37 ppm (bs, 1H, H-1′), ^13^C NMR: 108.2 ppm (C-1′)] and **16b** (β) [^1^H NMR: 5.36 ppm (d, *J*_1′,2′_ = 4.0 Hz, 1H, H-1′), ^13^C NMR: 95.6 ppm (C-1′)] in 89% yield (α:β = 2:1) (Scheme 3). Although the acceptor is also a thioglycoside, only the donor thioglycoside was activated in the NIS-catalyzed activation because of the higher reactivity of the furanosyl donor having benzyl protections [24–25].

**Scheme 4:**
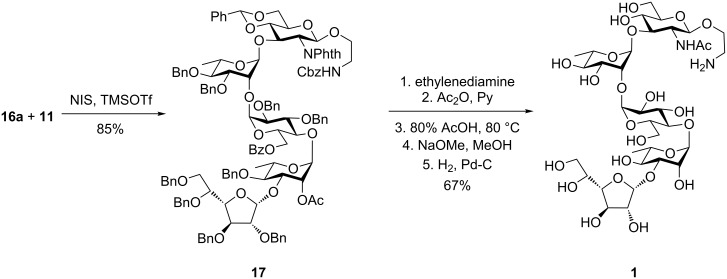
Synthesis of the target pentasaccharide **1**.

The mixture of α-linked and β-linked disaccharides was separated by flash chromatography to yield 59% pure α-linked disaccharide (**16a**) along with 30% β-linked disaccharide (**16b**). Compound **16a** was used for further glycosylation with the trisaccharide acceptor **11** to afford the protected pentasaccharide **17**. Once the protected pentasaccharide was at hand, the next challenge was the global deprotection to achieve the target molecule. First, the phthalimido group was removed using ethylenediamine [26] followed by acetylation with Ac_2_O in the presence of pyridine [27] to furnish the desired acetamido functionality. Next, the benzylidene group was hydrolyzed using 80% AcOH at 80 ºC [28]. Further, Zemplén de-*O*-acetylation using NaOMe in MeOH followed by hydrogenolysis in a ThalesNano continuous flow hydrogenation assembly using a 10% Pd-C cartridge [29]. After three cycles of hydrogenation, formation of the target pentasaccharide **1** was evident from the mass spectrum (Scheme 4).

## Conclusion

In conclusion, the pentasaccharide repeating unit of the *O*-specific polysaccharide of *Escherichia coli* O132 was accomplished through a convergent [3 + 2] strategy. The target pentasaccharide was obtained as its 2-aminoethyl glycoside that offers further possibilities for conjugation with suitable aglycons without hampering the stereochemistry of the reducing end sugar. The challenging stereoselective glycosylation with the galactofuranosyl unit was achieved through a chemoselective glycosylation approach depending on the higher reactivity of the furanose derivative over the pyranose system.

## Supporting Information

File 1 Detailed experimental descriptions for the preparation of all new compounds.

File 2 Copies of the ^1^H and ^13^C NMR spectra of all new compounds.
