# Supplementary material for: Chemical synthesis of the pentasaccharide repeating unit of the O-specific polysaccharide from Escherichia coli O132 in the form of its 2-aminoethyl glycoside
Source: Beilstein J Org Chem. 2019 Oct 28;15:2563–8. doi: 10.3762/bjoc.15.249 (PMC6839562; doi:10.3762/bjoc.15.249)
Supplement: File 1 — Detailed experimental descriptions for the preparation of all new compounds. [file Beilstein_J_Org_Chem-15-2563-s001.pdf]

BEILSTEIN JOURNAL OF ORGANIC CHEMISTRY

Supporting Information

for

Chemical synthesis of the pentasaccharide repeating unit of the *O*-specific polysaccharide from *Escherichia coli* O132 in the form of its 2-aminoethyl glycoside

Debasish Pal and Balaram Mukhopadhyay

Beilstein J. Org. Chem. **2019,** *15,* 2563–2568. doi:10.3762/bjoc.15.249

Detailed experimental descriptions for the preparation of all new compounds

License and Terms: This is a supporting information file under the terms of the Creative Commons Attribution License (http://creativecommons.org/licenses/by/4.0). Please note that the reuse, redistribution and reproduction in particular requires that the authors and source are credited.
The license is subject to the *Beilstein Journal of Organic Chemistry* terms and conditions: (https://www.beilstein-journals.org/bjoc)

Experimental section

General methods

All reactions were conducted under a dry nitrogen atmosphere. All solvents and reagents were dried prior to use according to standardized methods [1]. The commercially purchased reagents were used without any further purification unless mentioned otherwise. Dichloromethane was dried and distilled over P_2O_5. All reactions were monitored by thin-layer chromatography (TLC) on Silica-Gel 60-F254 with detection by UV lamp followed by charring after immersion in 10% ethanolic solution of H_2SO_4. Chromatographic separation was performed with silica gel 100–200 mesh and flash chromatography was performed with silica gel 230–400 mesh.

2-(Benzyloxycarbonylamino)ethyl 2-*O*-acetyl-3,4-di-*O*-benzyl-α-L-rhamnopyranosyl-(1→3)-4,6-*O*-benzylidene-2-deoxy-2-*N*-phthalimido-β-D-glucopyranoside (4): A mixture of known acceptor **2** (1.25 g, 2.2 mmol), donor **3** (1.3 g, 2.6 mmol) and MS 4Å (2.0 g) in dry CH_2Cl_2 (20 mL) was stirred under nitrogen for 5 min at −5 °C. Then NIS (764 mg, 3.4 mmol) was added followed by TMSOTf (0.94 µL, 0.52 mmol) and the mixture was stirred at −5 °C for another 15 min when TLC (*n*-hexane-EtOAc, 1.5:1) showed complete consumption of the acceptor. The reaction was quenched by Et_3N, filtered through a pad of Celite® and the filtrate was washed successively with aq. $Na_2S_2O_3$ (2 × 30 mL), saturated $NaHCO_3$ (2 × 30 mL) and H_2O (30 mL). The organic layer was collected, dried with Na_2SO_4 and filtered. The solvent was evaporated in vacuo and the residue was purified by flash chromatography using *n*-hexane/EtOAc (1:1) as

eluent to afford pure disaccharide **4** (1.9 g, 84%) as pale yellow amorphous mass. $[\alpha]_D^{25}$ = +42° (*c* 1.0, $CHCl_3$). ^1H NMR ($CDCl_3$, 500 MHz) δ: 7.62-7.15 (m, 24H, Ar*H*), 5.52 (s, 1H, C*H*Ph), 5.24 (d, 1H, $J_{1,2}$ 8.5 Hz, H-1), 5.01-4.90 (m, 3H, N*H*, CH_2CBz), 4.86 (bs, 1H, H-2'), 4.78 (d, 1H, 11.0 Hz, CH_2Ph), 4.59 (t, 1H, $J_{2,3}$ 9 Hz $J_{3,4}$ 9.5 Hz, H-3), 4.52 (s, 1H, H-1'), 4.46 (d, 2H, 11.5 Hz CH_2Ph), 4.40 (m, 2H, CH_2Ph, H-6a), 4.22 (t, 1H, $J_{1,2}$ 8.5 Hz $J_{2,3}$ 9.0 Hz, H-2), 3.88 (m, 1H, H-5'), 3.82-3.76 (m, 3H, H-3', O-CH_2), 3.66-3.59 (m, 3H, H-4, H-6b, CH_2N) , 3.27-3.20 (m, 3H, H-5, H-4', CH_2N), 1.78 (s, 3H, COCH_3), 0.76 (d, 3H, $J_{5',6'}$ 6.5 Hz, H-6'). ^{13}C NMR ($CDCl_3$, 125 MHz) δ 169.5 (*C*OCH_3), 156.1 (*C*OCbz), 138.5, 137.9, 136.9, 136.4, 134.2, 129.1, 128.4, 128.3, 128.2, 128.1, 127.9, 127.8, 127.7, 127.6, 127.5, 126.4 (Ar*C*), 101.9 (*C*HPh) 98.9 (*C*-1), 98.1 (C-1'), 80.3 (C-4), 79.9 (C-4'), 77.2 (C-3'), 75.1 (*C*H_2Ph), 74.9 (C-3), 71.4 (*C*H_2Ph), 69.2 (O-*C*H_2), 68.9 (C-2'), 68.8 (C-5), 68.5 (C-6), 67.9(C-5'), 66.5 (*C*H_2Ph), 56.3(C-2), 40.7 (*C*H_2N), 20.6 (CO*C*H_3), 17.16 (C-6'). HRMS calcd for $C_{53}H_{54}N_2O_{14}Na$ $(M+Na)^+$: 965.3473, found: 965.3468.

2-(Benzoyloxycarbonylamino)ethyl 3,4-di-*O*-benzyl-α-L-rhamnopyranosyl-(1→3)-4,6-*O*-benzylidine-2-deoxy-2-*N*-phthalimido-β-D-glucopyranoside (5): To a solution of compound **4** (750 mg, 0.70 mmol) in MeOH (10 mL), NaOMe in MeOH (0.5 M, 1 mL) was added and the solution was stirred at 25 ºC for 2 hours. Then the solution was neutralized with Dowex 50W X8 (H^+) resin, filtered and evaporated in vacco. The residue thus obtained was purified by column chromatography using eluent (*n*-hexane/ EtOAc = 2:1) to afford pure disaccharide acceptor **5** (661 mg, 91%) as white foam. $[\alpha]_D^{25}$ = +54° (*c* 1.0, $CHCl_3$). ^1H NMR ($CDCl_3$, 500 MHz) δ: 7.83-7.23 (m, 24H, Ar*H*), 5.54 (s, 1H, CHPh), 5.27 (d, 1H, $J_{1,2}$ 8.5 Hz, H-1), 5.02-4.92 (m, 3H, N*H*, CH_2CBz), 4.75 (d, 1H, 11.0 Hz,

CH_2Ph), 4.66 (t, 1H, $J_{2,3}$, $J_{3,4}$ 8.5 Hz, H-3), 4.63 (bs, 1H, H-1'), 4.57-4.48 (m, 3H, CH_2Ph), 4.39 (dd, 1H, $J_{5,6a}$ 4.0 Hz, $J_{6a,6b}$ 10.0 Hz, H-6a), 4.27 (t, 1H, $J_{1,2}$, $J_{2,3}$ 8.5 Hz, H-2), 3.94 (m, 1H, H-5'), 3.80 (m, 2H, H-6b, O-CH_2), 3.76 (dd, 1H, $J_{2',3'}$ 3.0 Hz, $J_{3',4'}$ 9.0 Hz, H-3'), 3.71 (bs, 1H, H-2′), 3.68-3.58 (m, 3H, H-4, H-5, O-CH_2), 3.25 (m, 3H, H-4', CH_2N), 2.28 (bs, 1H, O*H*), 0.79 (d, 3H, $J_{5',6'}$ 6.5 Hz, C-CH_3). ^{13}C NMR ($CDCl_3$, 125 MHz) δ: 156.1 (C*O*CBz), 138.5, 137.8, 136.8, 136.4, 134.4, 131.6, 129.0, 128.4 , 128.2, 128.1, 128.0, 127.9, 127.7, 127.6, 127.5, 126.3, 123.7 (Ar*C*), 101.9 (CHPh) 99.4 (*C*-1), 98.7 (*C*-1'), 80.6 (C-4), 79.7 (C-3'), 79.5 (C-4'), 75.0 (*C*H_2Ph), 73.9 (C-3), 71.8 (*C*H_2Ph), 69.1 (C-6), 68.5 (C-2′, O-*C*H_2), 67.6 (C-5'), 66.5 (C-5), 66.4 (*C*H_2Ph), 56.5 (C-2), 40.7 (*C*H_2N), 17.16 (C-6'). HRMS calcd for $C_{51}H_{52}N_2O_{13}Na$ $(M+Na)^+$: 923.3367, found: 923.3363.

Tolyl 2,3-di-*O*-benzyl-4-*O*-napthyl-6-*O*-*tert*-butyldiphenylsilyl-1-thio-β-D-glucopyranoside (7): To a solution of compound **6** (1.2 g ,1.70 mmol) in DMF (20 mL) stirred in an ice bath, NaH (122 mg, 5.12 mmol) was added followed by naphthyl bromide (488 mg, 2.21 mmol) and the mixture was stirred at 25 ºC for 8 hours after which TLC (*n*-hexane/EtOAc, 7:1) showed complete conversion of the starting material to a faster moving spot. DMF was evaporated under reduced pressure and the residue was stirred with H_2O (50 mL) for 30 minutes followed by extraction with EtOAc (2 × 25 mL). The organic layer was collected, dried with Na_2SO_4 and filtered. The solvent was evaporated in vacuo and the residue was purified by column chromatography using *n*-hexane/EtOAc (12:1) as eluent to afford pure compound **7** (1.2 g, 82%) as light yellow syrup. $[\alpha]_D^{25}$ = +48° (*c* 0.9, $CHCl_3$). ^{1}H NMR ($CDCl_3$, 500 MHz) δ: 7.71-6.92 (m, 31H, Ar*H*), 4.94 (d, 1H, 9.0 Hz, CH_2Ar), 4.84 (d, 1H, 8.0 Hz, CH_2Ar), 4.82 (d, 2H, 7.5 Hz, CH_2Ar), 4.77 (d, 1H,

10.0 Hz, CH_2Ar), 4.66 (d, 1H, 10.0 Hz, CH_2Ar), 4.56 (d, 1H, $J_{1,2}$ 9.5 Hz, H-1), 3.96-3.88 (m, 2H, H-6a, 6b), 3.77 (t, 1H, $J_{3,4}$, $J_{4,5}$ 9 Hz, H-4), 3.66 (t, 1H, $J_{2,3}$, $J_{3,4}$ 9.0 Hz, H-3), 3.46 (t, 1H, $J_{1,2}$, $J_{2,3}$ 9.0 Hz, H-2), 3.32 (m, 1H, H-5), 2.21 (s, 3H, $SC_6H_4CH_3$), 1.02 [s, 9H, C(CH_3)$_3$]. ^{13}C NMR ($CDCl_3$, 125 MHz) δ: 138.4, 138.2, 137.5, 135.8, 135.6, 135.1, 134.8, 133.4, 133.2, 132.9, 132.4, 130.1, 129.6, 128.4, 128.1, 127.9, 127.7, 127.6, 126.4, 126.0, 125.9, 125.8 (Ar*C*), 87.8 (C-1), 86.9 (C-3), 80.7 (C-2), 79.9 (C-5), 77.4 (C-4), 76.0 (*C*H_2Ar), 75.3 (*C*H_2Ar), 75.1 (*C*H_2Ar), 62.7 (C-6), 26.8, 26.5, 21.1 ($SC_6H_4CH_3$), 19.3. HRMS calcd for $C_{54}H_{56}O_5SSiNa$ (M+Na)$^+$: 867.3515, found: 867.3511.

Tolyl 2,3-di-*O*-benzyl-4-*O*-napthyl-1-thio-β-D-glucopyranoside (8): To a solution of compound **7** (1.1 g, 1.3 mmol) in dry THF (20 mL) at 0 ºC, 1 M TBAF in THF (3.9 mL, 3.9 mmol) was added and the mixture was warmed to 25 ºC and stirred for 3 hours. TLC (*n*-hexane/EtOAc, 5:1) showed complete conversion of the starting material to a slower running spot. Solvents were evaporated under reduced pressure, the residue was diluted with CH_2Cl_2 (20 mL) and it was successively washed with aq. saturated $NaHCO_3$ (2 × 25 mL) and brine (25 mL). The organic layer was separated, dried with Na_2SO_4 and filtered. The solvent was evaporated in vacuo and the residue was purified by column chromatography using *n*-hexane/EtOAc (2:1) as eluent to afford pure compound **8** (711 mg, 90%) as white foam. $[\alpha]_D^{25}$ = +62° (*c* 1.0, $CHCl_3$). ^{1}H NMR ($CDCl_3$, 500 MHz) δ: 7.85-7.14 (m, 21H, Ar*H*), 5.02 (d, 1H, 11.0 Hz, CH_2Ar), 4.96 (d, 2H, 10.0 Hz, CH_2Ar), 4.91 (d, 1H, 9.5 Hz, CH_2Ar), 4.84 (d, 1H, 11.5 Hz, CH_2Ar), 4.80 (d, 1H, 10.0 Hz, CH_2Ar), 4.70 (d, 1H, $J_{1,2}$ 8.0 Hz, H-1), 3.94 (dd, 1H, $J_{5,6a}$ 2.0 Hz, $J_{6a,6b}$ 12.0 Hz H-6a), 3.80-3.74 (m, 2H, H-3, H-6b), 3.65 (t, 1H, $J_{3,4}$, $J_{4,5}$ 6.0 Hz, H-4), 3.51 (t, 1H, $J_{1,2}$, $J_{2,3}$ 8.0 Hz, H-2), 3.43 (m,

1H, H-5), 2.36 (s, 3H, $SC_6H_4CH_3$). ^{13}C NMR ($CDCl_3$, 125 MHz) δ: 138.3, 138.0, 137.9, 135.3, 133.2, 132.9, 132.6, 129.8, 129.4, 128.4, 128.3, 128.2, 127.9, 127.8, 127.7, 127.6, 126.7, 126.1, 126.0, 125.8, 87.8 (C-1), 86.5 (C-3), 81.1 (C-2), 79.3 (C-5), 77.6 (C-4), 75.8 (*C*H_2Ar), 75.4 (*C*H_2Ar), 75.1 (*C*H_2Ar), 62.1 (C-6), 21.1 ($SC_6H_4CH_3$). HRMS calcd for $C_{38}H_{38}O_5SNa$ $(M+Na)^+$: 629.2338, found: 629.2333.

Tolyl 6-*O*-benzoyl-2,3-di-*O*-benzyl-4-*O*-napthyl-1-thio-β-D-glucopyranoside (9): To a solution of compound **8** (706 mg, 1.16 mmol) in dry CH_2Cl_2 (10 mL), pyridine (0.625 mL, 6.3 mmol) was added followed by BzCl (0.28 mL, 2.33 mmol) and DMAP (20 mg) and the solution was stirred at room temperature for 4 hours when TLC (*n*-hexane/EtOAc, 5:1) showed complete conversion of the starting material to a faster running spot. The reaction mixture was concentrated under reduced pressure and diluted with CH_2Cl_2 (15 mL) and washed successively with 1 N HCl (25 mL), $NaHCO_3$ (25 mL) and brine (25 mL). The organic layer was separated, dried by Na_2SO_4 and filtered. The solvent was evaporated in vacuo and the residue was purified by column chromatography using *n*-hexane/EtOAc 8:1 as eluent to afford pure compound **9** (747 mg, 90%) as white foam. $[\alpha]_D^{25}$ = +56° (*c* 1.1, $CHCl_3$). ^{1}H NMR ($CDCl_3$, 500 MHz) δ: 8.06-6.96 (m, 26H, Ar*H,*), 5.08 (d, 1H, 11.0 Hz, CH_2Ar), 5.02 (d, 2H, 10.5 Hz, CH_2Ar), 4.95 (d, 1H, 11.0 Hz, CH_2Ar), 4.86 (d, 1H, 11.0 Hz, CH_2Ar), 4.85 (d, 1H, 10.5 Hz, CH_2Ar), 4.78 (d, 1H, $J_{6a,6b}$ 12.0 Hz, H-6a), 4.70 (d, 1H, $J_{1,2}$ 9.0 Hz, H-1), 4.56 (dd, 1H, $J_{6a,6b}$ 12.0 Hz , $J_{5,6a}$ 2.5 Hz, H-6b), 3.86 (m, 1H, H-3), 3.76 (m, 2H, H-4, H-5), 3.46 (t, 1H, $J_{1,2}$, $J_{2,3}$ 9.0 Hz, H-2), 2.21 (s, 3H, $SC_6H_4CH_3$). ^{13}C NMR ($CDCl_3$, 125 MHz) δ: 165.9 (*C*OPh), 138.1, 137.9, 137.7, 134.8, 133.5, 133.1, 132.9, 130.1, 129.8, 129.6, 126.5, 129.3, 128.8, 128.4, 128.3, 128.2, 128.1, 127.8, 127.7, 127.5,

126.8, 126.0, 125.9, 125.8 (Ar*C*), 87.3 (C-1), 86.7 (C-3), 80.5 (C-2), 77.3 (C-5), 76.73 (C-4), 75.9 (*C*H_2Ar), 75.3 (*C*H_2Ar), 75.1 (*C*H_2Ar), 63.4 (C-6), 21.1 ($SC_6H_4CH_3$). HRMS calcd for $C_{45}H_{42}O_6SNa$ $(M+Na)^+$: 733.2600, found:733.2597.

2-(Benzoyloxycarbonylamino)ethyl 6-*O*-benzoyl-2,3-di-*O*-benzyl-4-*O*-napthyl-β-D-glucopyranosyl-(1→2)-3,4-di-*O*-benzyl-α-L-rhamnopyranosyl-(1→3)-4,6-*O*-benzylidene-2-deoxy-2-*N*-phthalimido-β-D-glucopyranoside (10): A solution of monosaccharide donor **9** (184 mg, 0.26 mmol), disaccharide acceptor **5** (210 mg, 0.23 mmol) and MS 4 Å (1.0 g) in dry CH_2Cl_2 (10 mL) was stirred under nitrogen for 5 minutes. Then, NIS (81 mg, 0.36 mmol) was added followed by TMSOTf (10 μL) and the mixture was stirred at −20 ºC for 1 hour when TLC (*n*-hexane/EtOAc, 1:1) showed complete consumption of the donor. The reaction was quenched by the addition of Et_3N, filtered through a pad of Celite® and the filtrate was washed successively with aq. $Na_2S_2O_3$ (2 × 15 mL), saturated $NaHCO_3$ (2 × 15 mL) and H_2O (20 mL). The organic layer was separated, dried by Na_2SO_4 and filtered. The solvent was evaporated in vacuo and the residue was purified by flash chromatography using *n*-hexane/EtOAc 2:1 as eluent to afford pure trisaccharide **10** (270 mg, 78%) as pale white amorphous mass along with unreacted disaccharide acceptor **5** (50 mg). $[\alpha]_D^{25}$ = +102° (*c* 0.8, $CHCl_3$). ^1H NMR ($CDCl_3$, 500 MHz) δ: 8.25-7.51 (m, 46H, ArH), 5.86 (s, 1H, C*H*-Ph) 5.57 (d, 1H, $J_{1,2}$ 10.0 Hz , H-1), 5.56 (s, 1H, N*H*), 5.28 (d, 1H, 12.0 Hz, CH_2Ar), 5.27 (d, 1H, 10.0 Hz, CH_2Ar), 5.23 (d, 1H, 9.0 Hz, CH_2Ar), 5.18 (d, 1H, 11.0 Hz, CH_2Ar), 5.12 (d, 1H, 11.0 Hz, CH_2Ar), 5.06 (d, 1H, 10.5 Hz, CH_2Ar), 5.00 (d, 1H, 11.0 Hz, CH_2Ar), 4.93 (s, 1H, H-1'), 4.92-4.84 (m, 4H, CH_2Ar, H-3), 4.69 (dd, 1H, $J_{5,6a}$ 4.5 Hz, $J_{6a,6b}$ 11.0 Hz, H-6a), 4.59 (m, 3H, CH_2Ar,

H-2,), 4.43 (d, 1H, 8.0 Hz, H-5''), 4.36 (d, 1H, 12.0 Hz, H-6a''), 4.27 (d, 1H, $J_{1'',2''}$ 1.5 Hz, H-1''), 4.25-4.10 (m, 6H, H-2', H-3', H-3'', H-6b, H-6b'', H-5'), 4.05-3.96 (m, 2H, H-4, O-CH_2), 3.94-3.91 (m, 2H, H-5, O-CH_2), 3.82 (t, 1H, $J_{3',4'}$ 9 Hz, $J_{4',5'}$ 9.0 Hz, H-4'), 3.76 (t, 1H, $J_{3'',4''}$ 9.5 Hz, $J_{4'',5''}$ 9.5 Hz H-4''), 3.51 (bs, 2H, CH_2N), (dd, 1H, $J_{1'',2''}$ 1.5 Hz, $J_{2'',3''}$ 9.0 Hz, H-2''),1.29 (d, 3H, $J_{5',6'}$ 6.5 Hz, H-6'). ^{13}C NMR ($CDCl_3$, 125 MHz) δ: 165.8 (*C*OPh), 156.1 (*C*OCbz), 138.5, 138.1, 138.0, 136.9, 136.4, 135.4, 134.2, 133.0, 132.9, 132.8, 130.7, 129.9, 129.4, 129.0, 128.4, 128.3, 128.2, 128.1, 128.0, 127.9, 127.8, 127.6, 127.4, 127.2, 127.0, 126.8, 126.3, 125.9, 125.8 (Ar*C*), 101.8 (*C*HPh), 98.9 (C-1), 97.4 (*C*-1'), 93.8 (*C*-1''), 81.3 (C-2'), 80.5 (C-5), 79.9 (C-4''), 79.6 (C-2''), 78.1 (C-4), 76.7 (C-4'), 75.5(*C*H_2Ar), 74.9 (*C*H_2Ar), 74.8 (*C*H_2Ar), 74.7 (*C*H_2Ar), 72.3 (*C*H_2O), 72.1 (*C*H_2Ar), 71.5 (C-3), 69.1 (C-5'), 68.6 (C-3'), 68.5 (C-3'', C-5''), 68.1 (C-6''), 66.6 (C-6), 66.5 (*C*H_2Ar), 62.9 (C-5'), 56.4 (C-2), 40.7 (*C*H_2N), 17.1 (C-6'). HRMS calcd for $C_{89}H_{86}N_2O_{19}Na$ $(M+Na)^+$: 1509.5722, found: 1509.5716.

2-(Benzoyloxycarbonylamino)ethyl 6-*O*-benzoyl-2,3-di-*O*-benzyl-β-D-glucopyranosyl-(1→2)-3,4-di-*O*-benzyl-α-L-rhamnopyranosyl-(1→3)-4,6-*O*-benzylidene-2-deoxy-2-*N*-phthalimido-β-D-glucopyranoside (11): To a solution of the trisaccharide **10** (150 mg, 0.1 mmol) in CH_2Cl_2/H_2O = 9:1 (10 mL), DDQ (26 mg, 0.11 mmol) was added and the mixture was stirred at 25 ^oC for 3 hours when TLC (*n*-hexane/EtOAc, 2:1) showed complete conversion to a slower moving spot. Then, the mixture was diluted with CH_2Cl_2 (10 mL) and washed successively with $NaHCO_3$ solution (2 × 15 mL) and brine (15 mL). The organic layer was separated, dried by Na_2SO_4 and filtered. The solvent was evaporated in vacuo and the residue was purified by flash

chromatography using *n*-hexane/EtOAc (2:1) as eluent to afford pure trisaccharide acceptor **11** (112 mg, 83%) as colourless syrup. $[\alpha]_D^{25}$ = +88° (*c* 0.9, $CHCl_3$). ^{1}H NMR ($CDCl_3$, 500 MHz) δ: 7.98-7.16 (m, 39H, ArH), 5.48 (s, 1H, C*H*Ph) 5.22 (d, 2H, $J_{1,2}$ 9.0 Hz , H-1,N*H*), 4.93 (d, 2H, 8.0 Hz, CH_2Ph), 4.84 (d, 1H, 12.0 Hz, CH_2Ph), 4.75 (d, 1H, 11.0 Hz, CH_2Ph), 4.62 (d, 1H, 11.0 Hz, CH_2Ph), 4.56 (s, 1H, H-1'), 4.55-4.46 (m, 4H, CH_2Ph, H-3), 4.22 (dd, 1H, $J_{6a,6b}$ 10.0 Hz , $J_{5,6a}$ 2.5 Hz, H-6a), 4.20 (t, 1H, $J_{1,2}$ 9.0 Hz, $J_{2,3}$ 9.0 Hz, H-2), 4.62 (s, 2H, CH_2Ph), 4.01 (d, 1H, 11.5 Hz, H-6a"), 3.95 (s, 1H, H-1"), 3.92-3.82 (m, 3H, H-5", H-5', H-6b"), 3.75-3.70 (m, 4H, H-3', H-2', H-6b, H-4), 3.65-3.59 (m, 3H, H-3", H-5, O-CH_2), 3.54 (m, 1H, O-CH_2), 3.37 (t, 1H, $J_{3',4'}$ 9.5 Hz, $J_{4',5'}$ 9.5 Hz H-4'), 3.29 (t, 1H, $J_{3'',4''}$ 9.0 Hz, $J_{4'',5''}$ 9.0 Hz H-4"), 3.20 (bs, 2H, NCH_2), (dd, 1H, $J_{1'',2''}$ 2 Hz, $J_{2'',3''}$ 9.0 Hz H-2"), 2.47 (bs, 1H, -OH), 0.83 (d, 3H, $J_{5',6'}$ 6.0 Hz, H-6').^{13}C NMR ($CDCl_3$, 125 MHz) δ: 166.6 (*C*OPh), 156.1 (*C*OCbz), 138.5(2), 138.2, 137.9, 136.9, 136.4, 134.3, 133.2, 130.9, 129.9, 129.7, 129.2, 128.4, 128.3, 128.2, 128.1, 128.0, 127.9, 127.7, 127.6, 127.4, 127.1, 126.3 (Ar*C*), 101.8 (*C*HPh), 98.9 (C-1), 97.3 (C-1'), 94.2 (C-1"), 80.5 (C-3"), 80.2 (C-5), 80.1 (C-4'), 79.1 (C-2"), 78.2 (C-3'), 75.2 (*C*H_2Ph), 74.9 (*C*H_2Ph), 74.5 (*C*H_2Ph), 72.5 (*C*-2'), 72.1 (C-3), 71.4 (*C*H_2Ph), 69.6 (C-5"), 69.2 (O*C*H_2), 69.1 (C-4"), 68.6 (C-5'), 68.5 (C-6), 66.6 (C-4), 66.5(*C*H_2Ph), 63.2(C-6"), 56.5 (C-2), 40.7 (*C*H_2N), 17.3 (C-6'). HRMS calcd for $C_{78}H_{78}N_2O_{19}Na$ $(M+Na)^+$: 1369.5096, found:1369.5090.

***p*-Tolyl 2,3,5,6-tetra-*O*-benzyl-1-thio-β-D-galactofuranoside (14)**: To a solution of the known galactofuranoside derivative **12** in MeOH (20 mL), NaOMe in MeOH (0.5 M, 2 mL) was added and the solution was stirred at room temperature for 1 hours till completion of the reaction was confirmed by TLC (*n*-hexane/EtOAc = 1:1). Excess NaOMe was

neutralized with Dowex 50W X8 (H^+) resin, filtered and the solvents were evaporated in vacuo to give compound **13** which was used further without any purification. Compound **13** (1.44 g, 5.03 mmol) was dissolved in DMF (25 mL) and stirred in ice bath for 5 minutes. NaH (1.44 g, 60 mmol, 50% in mineral oil) was added followed by BnBr (3 mL, 26 mmol) and the mixture was stirred at 25 ºC for 8 hours when TLC (*n*-hexane/EtOAc, 8:1) confirmed complete reaction. DMF was evaporated under reduced pressure, H_2O (50 mL) was added and stirred for 30 minutes. Then the compound was extracted with EtOAc (2 × 25 mL). The organic layer was separated, dried with Na_2SO_4 and filtered. The solvent was evaporated in vacuo and the residue was purified by flash chromatography using *n*-hexane/EtOAc 14:1 as eluent to afford pure galactofuranose donor **14** (2.9 g, 89%) as pale yellow syrup. $[\alpha]_D^{25}$ = +132° (*c* 1.0, $CHCl_3$). ^{1}H NMR ($CDCl_3$, 500 MHz) δ: 7.40-7.01 (m, 24H, Ar*H*), 5.56 (d, 1H, 1.5 Hz, H-1), 4.72 (d, 1H, 11.5 Hz, CH_2Ph), 4.66 (d, 1H, 12.0 Hz, CH_2Ph), 4.53 (d, 4H, 13.0 Hz, CH_2Ph), 4.46 (d, 1H, 7.0 Hz, CH_2Ph), 4.37 (dd, 1H, $J_{3,4}$ 7.5 Hz $J_{4,5}$ 3.5 Hz, H-4), 4.34 (d, 1H, 12.0 Hz, CH_2Ph), 4.13-4.08 (m, 2H, H-2, H-3), 3.85 (m, 1H, H-5), 3.75-3.67 (m, 2H, H-6a, H-6b), 2.33 (s, 3H, $SC_6H_4CH_3$). ^{13}C NMR ($CDCl_3$, 125 MHz) δ: 138.3, 138.2, 137.7, 137.4, 137.2, 132.0, 130.8, 129.5, 128.3, 128.2, 127.9, 127.8, 127.7, 127.5 (Ar*C*), 90.2 (*C*-1), 88.5 (*C*-2), 82.8 (*C*-3), 80.5 (*C*-4), 77.3 (*C*-5), 73.4 (*C*H_2Ph), 73.2 (*C*H_2Ph), 72.1 (*C*H_2Ph), 71.9 (*C*H_2Ph), 70.7 (C-6), 21.1 (SC_6H_4*C*H_3). HRMS calcd for $C_{41}H_{42}O_5SNa$ $(M+Na)^+$: 669.2651, found: 669.2547.

***p*-Tolyl 2,3,5,6-tetra-*O*-benzyl-α-D-galactofuranosyl-(1→3)-2-*O*-acetyl-4-*O*-benzyl-1-thio-α-L-rhamnopyranoside (16a)**: A mixture of galactofuranoside donor **14** (940 mg, 1.45 mmol), known rhamnose acceptor **15** (487 mg, 1.21 mmol) and MS 4 Å (3 g) in dry

CH_2Cl_2 (30 mL) was stirred under a nitrogen atmosphere for 10 minutes at −40 ºC. NIS (428 mg, 1.9 mmol) was added followed by TMSOTf (53 μl, mmol) and allowed to stir at the same temperature for 30 minutes when TLC (*n*-hexane-EtOAc, 8:1) showed complete consumption of the donor. The reaction was quenched by the addition of Et_3N and filtered through a pad of Celite®. The filtrate was washed successively with aq. $Na_2S_2O_3$ (2 × 30 mL), saturated $NaHCO_3$ (2 × 30 mL) and H_2O (30 mL). The organic layer was separated, dried by Na_2SO_4 and filtered. The solvent was evaporated in vacuo and the residue was purified by flash chromatography using *n*-hexane/EtOAc (9 :1) as eluent to afford the α-disaccharide **16a** (450 mg, 59%) and β-disaccharide **16b** (228 mg, 30%) as pale yellow amorphous mass. $[\alpha]_D^{25}$ = +84° (*c* 0.9, $CHCl_3$). ^1H NMR ($CDCl_3$, 500 MHz) δ: 7.35-7.07 (m, 34H, ArH), 5.44 (dd, 1H, $J_{1,2}$ 1.5 Hz, $J_{2,3}$ 3.0 Hz, H-2), 5.37 (bs, 1H, H-1'), 5.32 (d, 1H, $J_{1,2}$ 1.5 Hz , H-1), 4.90 (d, 1H, 11.0 Hz, CH_2Ph), 4.76 (d, 1H, 12.0 Hz, CH_2Ph), 4.66 (d, 1H, 11.5 Hz, CH_2Ph), 4.59 (d, 1H, 12.0 Hz, CH_2Ph), 4.54 (d, 1H, 7.0 Hz, CH_2Ph), 4.52 (d, 1H, 7.5 Hz, CH_2Ph), 4.40 (d, 1H, 8.5 Hz, CH_2Ph), 4.39 (d, 1H, 8.0 Hz, CH_2Ph), 4.30 (d, 2H, 12.0 Hz, CH_2Ph), 4.22 (m, 1H, H-5), 4.15 (dd, 1H, $J_{2,3}$ 3.0 Hz, $J_{3,4}$ 6.5 Hz, H-3), 4.09 (dd, 1H, $J_{3',4'}$ 9.5 Hz, $J_{4',5'}$ 4.0 Hz H-4'), 4.04 (m, 2H, H-2', H-3'), 3.80 (m, 1H, H-5'), 3.77-3.71 (m, 2H, H-6a', 6b'), 3.52 (t, 1H, $J_{3,4}$ 6.5 Hz $J_{4,5}$ 6.5 Hz, H-4), 2.29 (s, 3H, $SC_6H_4CH_3$), 2.01 (s, 3H, CH_3CO), 1.29 (d, 3H, $J_{5,6}$ 6.5 Hz, H-6). ^{13}C NMR ($CDCl_3$, 125 MHz) δ: 170.2 (CH_3*C*O), 138.5, 138.3, 137.9, 137.7, 137.6, 132.2, 130.2, 129.8, 128.5, 128.4, 128.3, 128.2, 128.1, 127.9, 127.8, 127.6, 127.4, 127.2 (Ar*C*), 108.2 (*C*-1'), 88.7 (C-2'), 86.1 (C-1), 82.9 (C-3'), 82.2 (C-3), 80.1 (C-4), 77.4 (C-4'), 76.3 (C-5'), 75.1(*C*H_2Ph), 74.3 (C-2), 73.4 (*C*H_2Ph), 73.2 (*C*H_2Ph), 71.9 (*C*H_2Ph), 71.5 (C-6', *C*H_2Ph), 68.9 (C-5), 21.1

($SC_6H_4CH_3$), 20.9 (CH_3CO), 17.8 (C-6). HRMS calcd for $C_{56}H_{60}O_{10}SNa$ $(M+Na)^+$: 947.3805, found: 947.3801.

***p*-Tolyl 2,3,5,6-tetra-*O*-benzyl-β-D-galactofuranosyl-(1→3)-2-*O*-acetyl-4-*O*-benzyl-1-thio-α-L-rhamnopyranoside (16b)**: $[\alpha]_D^{25}$ = +72° (*c* 1.0, $CHCl_3$). ^1H NMR ($CDCl_3$, 500 MHz) δ: 7.40-7.08 (m, 34H, ArH), 5.65 (dd, 1H, $J_{1,2}$ 1.5 Hz, $J_{2,3}$ 4.0 Hz, H-2), 5.36 (d, 1H, $J_{1,2}$ 4.0 Hz H-1'), 5.28 (d, 1H, $J_{1,2}$ 1.5 Hz , H-1), 4.95 (d, 1H, 11.5 Hz, CH_2Ph), 4.74 (d, 1H, 11.5 Hz, CH_2Ph), 4.69 (d, 1H, 11.5 Hz, CH_2Ph), 4.66-4.62 (m, 3H, CH_2Ph), 4.49 (dd, 1H, $J_{2,3}$ 4.0 Hz, $J_{3,4}$ 9.5 Hz, H-3), 4.44 (d, 2H, 11.5 Hz, CH_2Ph), 4.29 (d, 2H, 6 Hz, CH_2Ph), 4.23-4.13 (m, 3H, H-5, H-3', H-2'), 4.40 (t, 1H, $J_{3',4'}$ 7.5 Hz $J_{4',5'}$ 7.0 Hz, H-4'), 3.76 (m, 1H, H-5'), 3.53 (t, 1H, $J_{3,4}$ 9.5 Hz $J_{4,5}$ 9.5 Hz, H-4), 3.34 (dd, 1H, $J_{5,6a'}$ 3.5 Hz, $J_{6a',6b'}$ 10.5 Hz, H-6a'), 3.24 (dd, 1H, $J_{5,6b'}$ 5.5 Hz, $J_{6a',6b'}$ 10.5 Hz, H-6b'), 2.29 (s, 3H, $SC_6H_4CH_3$), 1.90 (s, 3H, CH_3CO), 1.25 (d, 3H, $J_{5,6}$ 6.5 Hz, H-6). ^{13}C NMR ($CDCl_3$, 125 MHz) δ: 170.2 (CH_3CO), 139.3, 139.1, 138.4, 138.3, 137.9, 137.8, 132.1, 130.2, 129.8, 128.4, 128.3, 128.2, 128.1, 128.0, 127.9, 127.8, 127.6, 127.5, 127.4, 127.3, 127.1 (Ar*C*), 95.6 (*C*-1'), 86.2 (C-1), 84.3 (C-5), 81.3 (C-3'), 81.2 (C-4'), 80.7 (C-5'), 79.0 (C-4), 75.1 (CH_2Ph), 73.2 (C-3), 72.5 (CH_2Ph), 71.8 (2 CH_2Ph), 71.6 (CH_2Ph), 69.7 (C-2), 69.5 (C-6'), 69.3 (C-2'), 21.1 ($SC_6H_4CH_3$), 20.1 (CH_3CO), 17.8 (C-6). HRMS calcd for $C_{56}H_{60}O_{10}SNa$ $(M+Na)^+$: 947.3805, found: 947.3802.

2-(Benzyloxycarbonylamino)ethyl 2,3,5,6-tetra-*O*-benzyl-β-D-galactofuranosyl-(1→3)-2-*O*-acetyl-4-*O*-benzyl-α-L-rhamnopyranosyl-(1→4)-6-*O*-benzoyl-2,3-di-*O*-benzyl-β-D-glucopyranosyl-(1→2)-3,4-di-*O*-benzyl-α-L-rhamnopyranosyl-(1→3)-4,6-

***O*-benzylidene-2-deoxy-2-*N*-phthalimido-β-D-glucopyranoside (17)**: A mixture of disaccharide donor **16** (71 mg, 0.08 mmol), trisaccharide acceptor (86 mg, 0.06 mmol) and MS 4 Å (1 g) in dry CH_2Cl_2 (10 mL) was stirred under nitrogen atmosphere for 5 minutes at −5 ºC. NIS (23 mg, 0.10 mmol) was added followed by TMSOTf (3 μL, 0.015 mmol) was added and the mixture was allowed to stir for 1 hour at the same temperature when TLC (*n*-hexane/EtOAc, 2:1) showed complete consumption of the donor. The reaction was quenched by the addition of Et_3N and the mixture was filtered through a pad of Celite®. The filtrate was diluted with CH_2Cl_2 (10 mL) and washed successively with aq. $Na_2S_2O_3$ (2 × 15 mL), saturated $NaHCO_3$ (2 × 15 mL) and H_2O (20 mL). The organic layer was separated, dried by Na_2SO_4 and filtered. The solvent was evaporated in vacuo and the residue was purified by flash chromatography using *n*-hexane/EtOAc (2:1) as eluent to afford pure protected pentasaccharide **17** (116 mg, 85%) as pale yellow foam. $[\alpha]_D^{25}$ = +34° (*c* 0.8, $CHCl_3$). ^1H NMR ($CDCl_3$, 500 MHz) δ: 7.37-7.09 (m, 64H, Ar*H*), 5.47 (s, 1H, C*H*Ph), 5.23 (bs, 1H, H-1''''), 5.08 (d, 1H, $J_{1,2}$ 10.0 Hz , H-1), 5.08 (dd, 1H, $J_{1''',2'''}$ 1.5 Hz, $J_{2''',3'''}$ 3.0 Hz, H-2'''), 4.98-4.86 (m, 2H, CH_2Ph), 4.83 (s, 1H, N*H*), 4.80 (bs, 1H, H-1'), 4.77 (d, 2H, 6.5 Hz, CH_2Ph), 4.72 (d, 1H, 6.0 Hz, CH_2Ph), 4.68 (d, 1H, 10.0 Hz, CH_2Ph), 4.58 (d, 2H, 11.0 Hz, CH_2Ph), 4.54 (d, 1H, 12.0 Hz, CH_2Ph), 4.51-4.48 (m, 5H, CH_2Ph, H-3, H-1'''), 4.37 (d, 1H, 12.0 Hz, CH_2Ph), 4.37 (d, 2H, 14.0 Hz, CH_2Ph) 4.33-4.26 (m, 4H, CH_2Ph), 4.24 (d, 2H, 9.0 Hz, CH_2Ph, H-2'), 4.20-4.16 (m, 2H, H-2, H-6a''), 4.09-4.06 (m, 2H, H-6a, H-5''''), 4.02-3.92 (m, 6H, H-6b, H-5', H-5'', H-5, H-3''', H-4'''', H-2''''), 3.78 (m, 1H, H-5'''), 3.74 (d, 1H, $J_{1'',2''}$ 1.5 Hz, H-1''), 4.72-4.53 (m, 10H, H-3', H-3'', H-3'''', H-6a'''', H-6b'''', H-4, H-4'', H-6b'', O-CH_2), 3.37 (t, 1H, $J_{3''',4'''}$ 9.5 Hz, $J_{4''',5'''}$ 9.5 Hz, H-4'''), 3.30 (t, 1H, $J_{3',4'}$ 9.5 Hz, $J_{4',5'}$ 9.5 Hz H-4'), 3.19 (bs, 2H, CH_2N), 2.95 (dd, 1H, $J_{1'',2''}$ 3.5 Hz, $J_{2'',3''}$

9.0 Hz, H-2''), 1.81 1.90 (s, 3H, CH_3CO), 0.95 (d, 3H, $J_{5',6'}$ 6.5 Hz, H-6'), 0.78 (d, 3H, $J_{5''',6'''}$ 6.5 Hz, H-6'''), ^{13}C NMR ($CDCl_3$, 125 MHz) δ: 169.9 (CH_3CO), 165.5 (*C*OPh), 156.2 (*C*OCbz), 139.2, 138.7, 138.6, 138.5, 138.3, 138.1, 138.0, 137.8, 137.5, 136.9, 136.4, 135.8, 134.3, 134.3, 132.9, 130.9, 130.2, 129.8, 129.7, 128.9, 128.4, 128.3, 128.2, 128.1, 128.0, 127.9, 127.8, 127.7, 127.6, 127.5, 127.4, 127.3, 127.2, 127.1, 127.3, (Ar*C*), 108.3 (C-1''''), 101.8 (*C*HPh), 98.9 (C-1), 97.2 (*C*-1'), 97.1 (C-1'''), 93.8 (*C*-1''), 88.9 (C-2''''), 82.5 (C-4''''), 82.3 (C-5''''), 80.4 (C-4), 80.1 (C-2''), 80.0 (C-4'''), 79.9 (C-4'), 79.0 (C-3''''), 77.8 (C-3''), 77.7 (C-3'''), 76.6 (C-3'), 75.5 (*C*H_2Ph), 75.0 (2 *C*H_2Ph, C-2'), 74.9 (*C*H_2Ph), 74.5 (C-3), 74.2 (C-4''), 73.1 (2 *C*H_2Ph), 73.0 (*C*H_2Ph), 72.3 (C-2'''), 72.0 (*C*H_2Ph), 71.9 (*C*H_2Ph), 71.5 (C-6), 71.4 (C-6''''), 69.1 (C-5'), 68.6 (C-5''', C-5''), 68.1 (C-5), 66.6 (*C*H_2Ph), 66.5(O-*C*H_2), 62.4 (C-6''), 56.4 (C-2), 41.3 (*C*H_2N), 20.9 (*C*H_3CO), 17.7 (C-6'), 17.3 (C-6'''). HRMS calcd for calcd for $C_{127}H_{130}N_2O_{29}$ Na $(M+Na)^+$: 2169.8657, found: 2169.8651.

2-aminoethyl β-D-galactofuranosyl-(1→3)-α-L-rhamnopyranosyl-(1→4)-β-D-glucopyranosyl-(1→2)-α-L-rhamnopyranosyl-(1→3)-2-acetamido-2-deoxy-β-D-glucopyranoside (1): To a solution of the compound **17** (110 mg, 0.051 mmol) in 1-butanol (5 mL), ethylenediamine (0.1 mL) was added and the mixture was stirred at 110 ºC for 24 hours till TLC (CH_2Cl_2/MeOH; 10:1) showed complete conversion. The solvents were evaporated under reduced pressure, the residue was dissolved in pyridine (5 mL) followed by addition of Ac_2O (2 mL) and allowed to stir at 50 ºC for 5 hours. After evaporation of the solvents and co-evaporation with toluene to remove excess pyridine, the residue was dissolved in 80% aqueous AcOH (5 mL) and stirred at 80 ºC for 2 hours. The solvents were evaporated and the residue was dissolved in MeOH (7 mL). NaOMe

in MeOH (0.5 M, 1 mL) was added and the solution was stirred at room temperature for 5 hours. Then, it was neutralized with DOWEX 50W H^+ resin and filtered. The filtrate was evaporated under reduced pressure and the residue was dissolved in MeOH (50 mL). This methanolic solution was passed through ThalesNano continuous flow hydrogenation assembly with a 10% Pd-C cartridge for three consecutive rounds untill complete hydrogenolysis. The solvents were evaporated and the residue was purified by size exclusion column chromatography on Sephadex G-25 using water as the eluent to afford compound **1** (30 mg, 67%) as off white amorphous mass. $[\alpha]_D^{25}$ = +48° (*c* 0.7, H_2O). ^1H NMR (CD_3OD, 500 MHz) δ: 5.19 (s, 1H, H-1′′′′), 4.99 (s, 1H, H-1′′′), 4.81 (s, 1H, H-1′), 4.77 (d, 1H, $J_{1,2}$ 7.5 Hz, H-1), 4.72 (d, 1H, $J_{1'',2''}$ 3.0 Hz, H-1′′), 1.94 (s, 3H, NHCOCH_3), 1.32 (d, 3H, *J* 6.0 Hz, C-6′′′), 1.29 (d, 3H, *J* 6.5 Hz, C-6′). ^{13}C NMR (CD_3OD, 125MHz) δ: 173.9 (CH_3*C*O), 111.2 (C-1''''), 102.6 (C-1), 101.8 (*C*-1'), 100.2 (C-1'''), 99.5 (*C*-1''), 85.6, 82.6, 81.1, 79.9, 79.5, 78.9, 78.8, 78.1, 75.9, 73.7, 72.6, 72.3, 71.7, 70.6, 69.8, 65.7, 64.4, 64.3, 64.2, 62.6, 62.5, 61.6, 58.4, 56.9, 43.9 (*C*H_2N), 23.7 (*C*H_3CO), 17.9 (C-6'), 17.8 (C-6'''). HRMS calcd for $C_{34}H_{60}N_2O_{24}Na$ $(M+Na)^+$: 903.3434, found: 903.3431.

Reference

[1] Perrin, D. D.; Amarego, W. L.; Perrin, D. R. *Purification of Laboratory Chemicals, Pergamon, London,* **1996.**
