# Supplementary material for: Chemical synthesis of the pentasaccharide repeating unit of the O-specific polysaccharide from Escherichia coli O132 in the form of its 2-aminoethyl glycoside
Source: Beilstein J Org Chem. 2019 Oct 28;15:2563–8. doi: 10.3762/bjoc.15.249 (PMC6839562; doi:10.3762/bjoc.15.249)

BEILSTEIN JOURNAL OF ORGANIC CHEMISTRY

Supporting Information

for

Chemical synthesis of the pentasaccharide repeating unit of the *O*-specific polysaccharide from *Escherichia coli* O132 in the form of its 2-aminoethyl glycoside

Debasish Pal and Balaram Mukhopadhyay

Beilstein J. Org. Chem. **2019,** *15,* 2563–2568. doi:10.3762/bjoc.15.249

Copies of the ^{1}H and ^{13}C NMR spectra of all new compounds

License and Terms: This is a supporting information file under the terms of the Creative Commons Attribution License (http://creativecommons.org/licenses/by/4.0). Please note that the reuse, redistribution and reproduction in particular requires that the authors and source are credited.
The license is subject to the *Beilstein Journal of Organic Chemistry* terms and conditions: (https://www.beilstein-journals.org/bjoc)

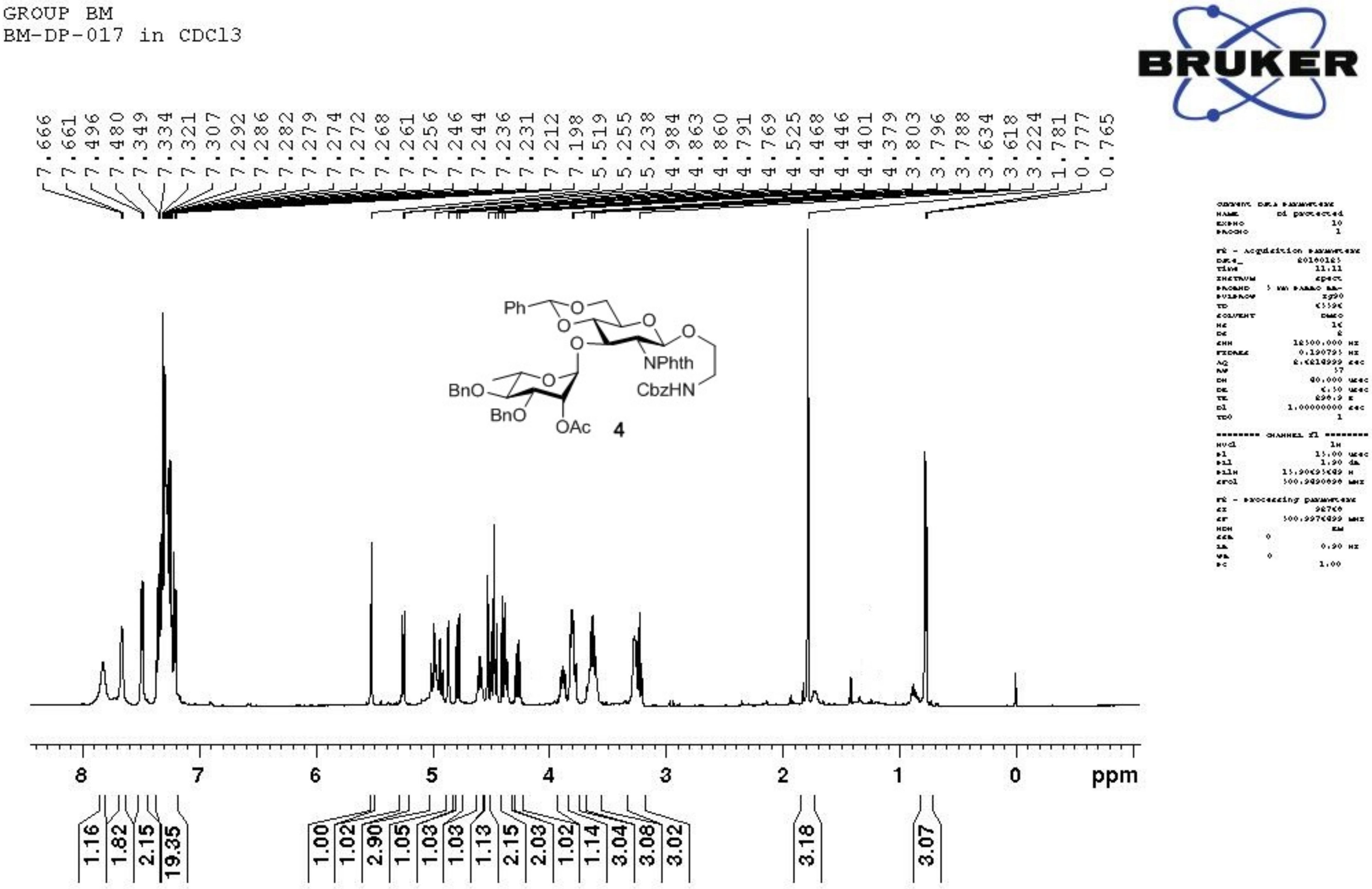
GROUP BM
BM-DP-017 in CDCl3
7.666 7.661 7.496 7.480 7.349 7.334 7.321 7.307 7.292 7.286 7.282 7.279 7.274 7.272 7.268 7.261 7.256 7.246 7.244 7.236 7.231 7.212 7.198 5.519 5.255 5.238 4.984 4.863 4.860 4.791 4.769 4.525 4.468 4.446 4.401 4.379 3.803 3.796 3.788 3.634 3.618 3.224 1.781 0.777 0.765
Ph
NPhth
CbzHN
BnO
BnO
OAc
4
8 7 6 5 4 3 2 1 0 ppm
1.16 1.82 2.15 19.35 1.00 1.02 2.90 1.05 1.03 1.03 1.13 2.15 2.03 1.02 1.14 3.04 3.08 3.02 3.18 3.07

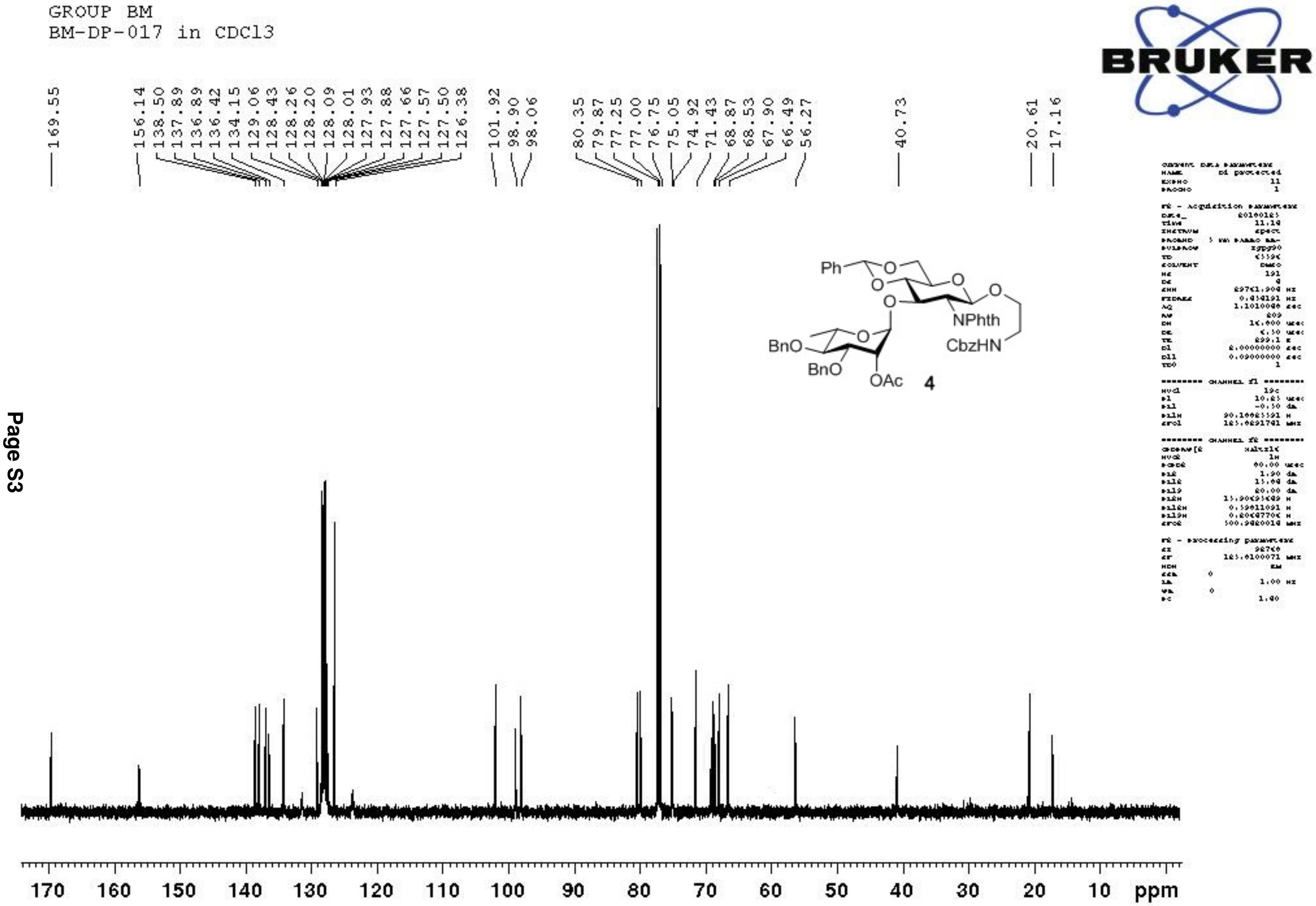
GROUP BM
BM-DP-017 in CDCl3
169.55
156.14
138.50
137.89
136.89
136.42
134.15
129.06
128.43
128.26
128.20
128.09
128.01
127.93
127.88
127.66
127.57
127.50
126.38
101.92
98.90
98.06
80.35
79.87
77.25
77.00
76.75
75.05
74.92
71.43
68.87
68.53
67.90
66.49
56.27
40.73
20.61
17.16
Ph
O
NPhth
CbzHN
BnO
OAc
ppm

4

GROUP BM
BM-DP-068B in CDCl3

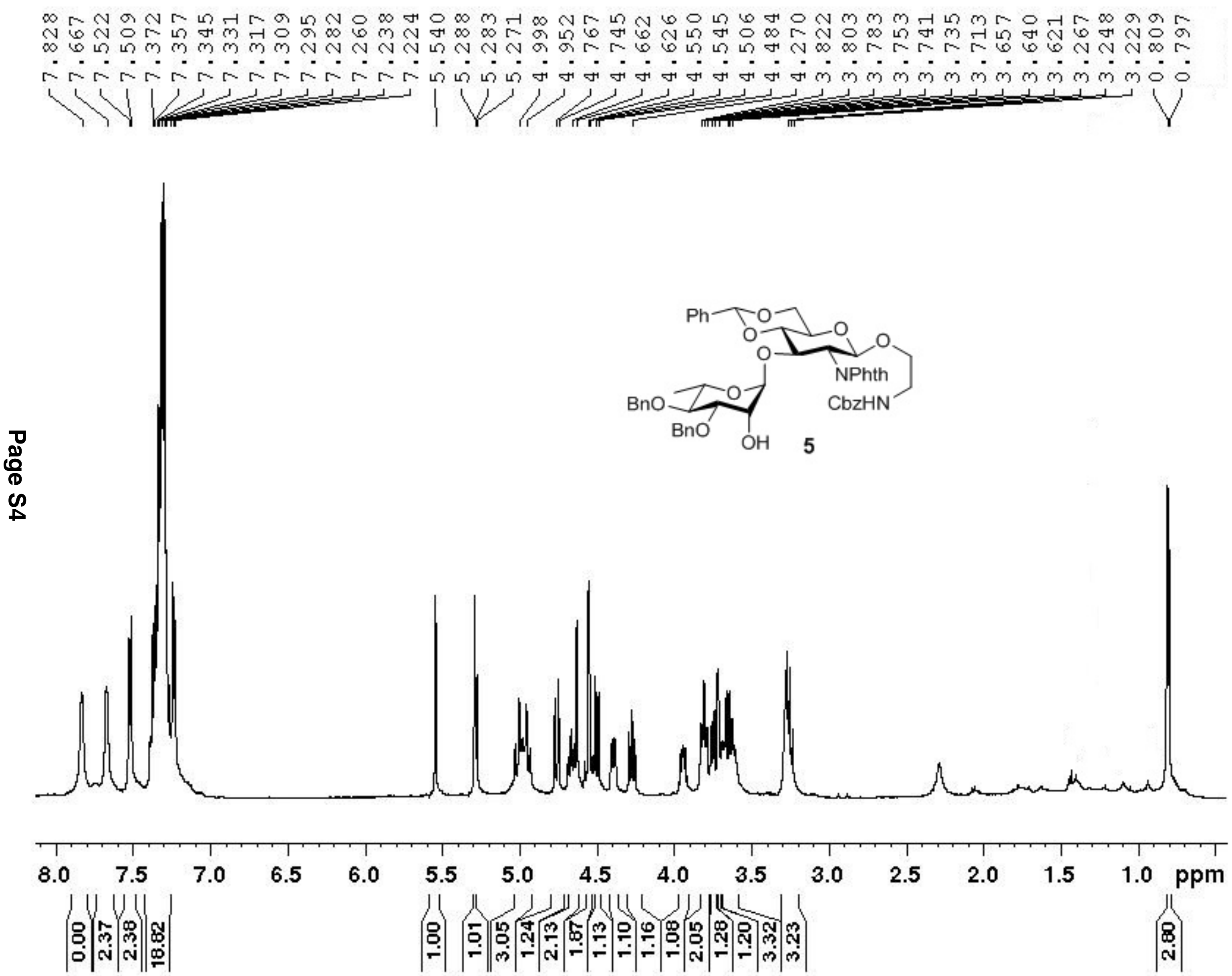

5

```
Current Data Parameters
NAME              DI ACCEPTOR
EXPNO                      20
PROCNO                      1

F2 - Acquisition Parameters
Date_                20180730
Time                    11.45
INSTRUM   Bruker_default_av500
PROBHD     5 mm PABBO BB/
PULPROG                  zg30
TD                      66560
SOLVENT                 CDCl3
NS                         16
DS                          2
SWH                 10000.000 Hz
FIDRES               0.150240 Hz
AQ                  3.3280001 sec
RG                         36
DW                     50.000 usec
DE                       6.50 usec
TE                      298.2 K
D1                 1.00000000 sec
TD0                         1

-------- CHANNEL f1 --------
SFO1              500.3430898 MHz
NUC1                       1H
P1                      17.50 usec
PLW1              10.00000000 W

F2 - Processing parameters
SI                     131072
SF                500.3400125 MHz
WDW                        EM
SSB      0
LB                       0.60 Hz
GB       0
PC                       1.00
```

GROUP BM
BM-DP-068B in CDCl3

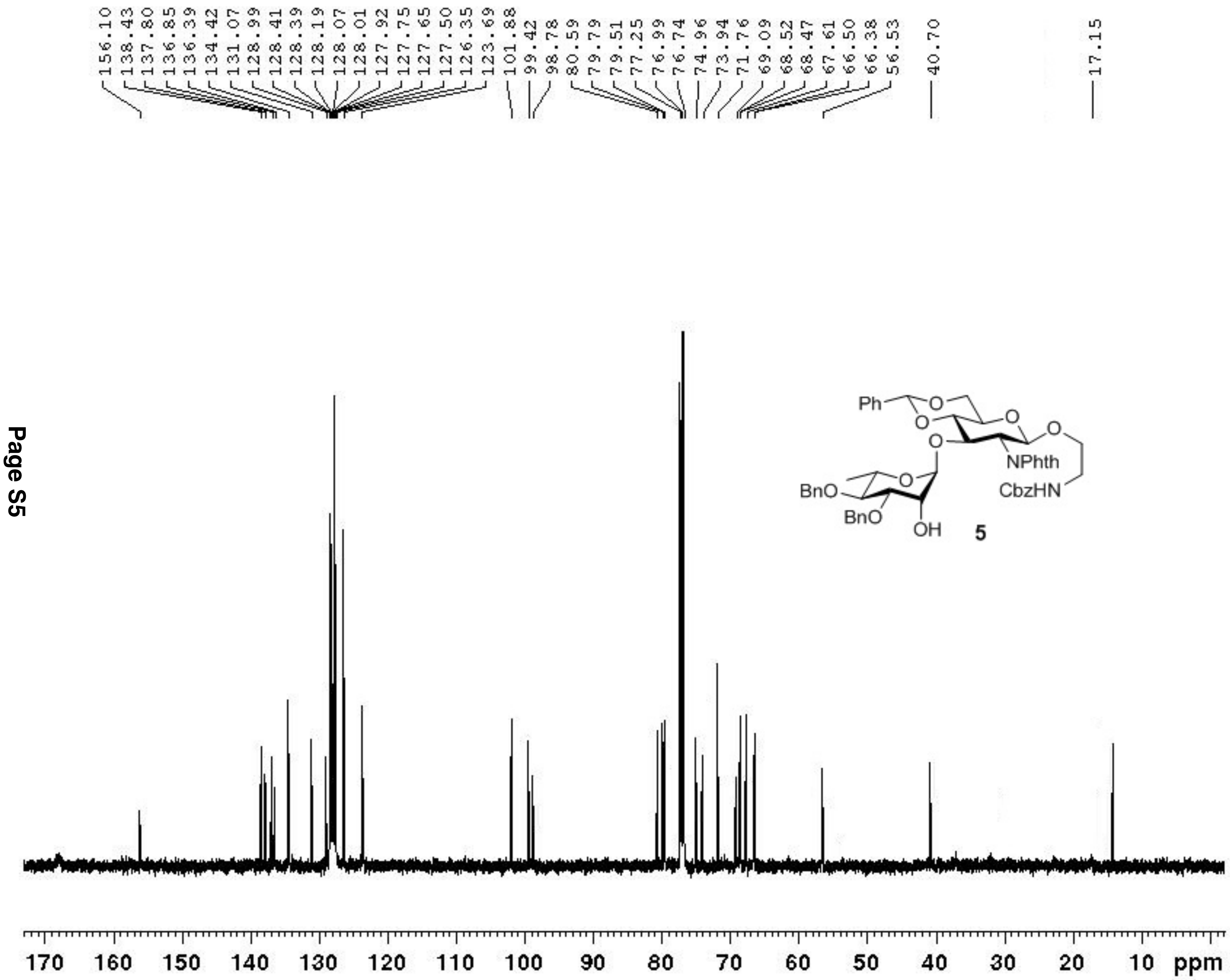

BRUKER

```
Current Data Parameters
NAME          DI ACCEPTOR
EXPNO                  21
PROCNO                  1

F2 - Acquisition Parameters
Date_            20180730
Time                11.50
INSTRUM   Bruker_default_av500
PROBHD    5 mm PABBO BB/
PULPROG            zgpg30
TD                  65536
SOLVENT             CDCl3
NS                    181
DS                      4
SWH            43859.648 Hz
FIDRES          0.669245 Hz
AQ             0.7471104 sec
RG                    203
DW                 11.400 usec
DE                   6.50 usec
TE                  298.5 K
D1            2.00000000 sec
D11           0.03000000 sec
TD0                     1

-------- CHANNEL f1 --------
SFO1         125.8294646 MHz
NUC1                  13C
P1                  11.50 usec
PLW1         66.00000000 W

-------- CHANNEL f2 --------
SFO2         500.3420014 MHz
NUC2                   1H
CPDPRG[2          waltz16
PCPD2               80.00 usec
PLW2         10.00000000 W
PLW12         0.47852001 W
PLW13         0.24068999 W

F2 - Processing parameters
SI                  32768
SF           125.8106086 MHz
WDW                    EM
SSB          0
LB                   1.00 Hz
GB           0
PC                   1.40
```

GROUP BM
BM-DP-063 in CDCl3

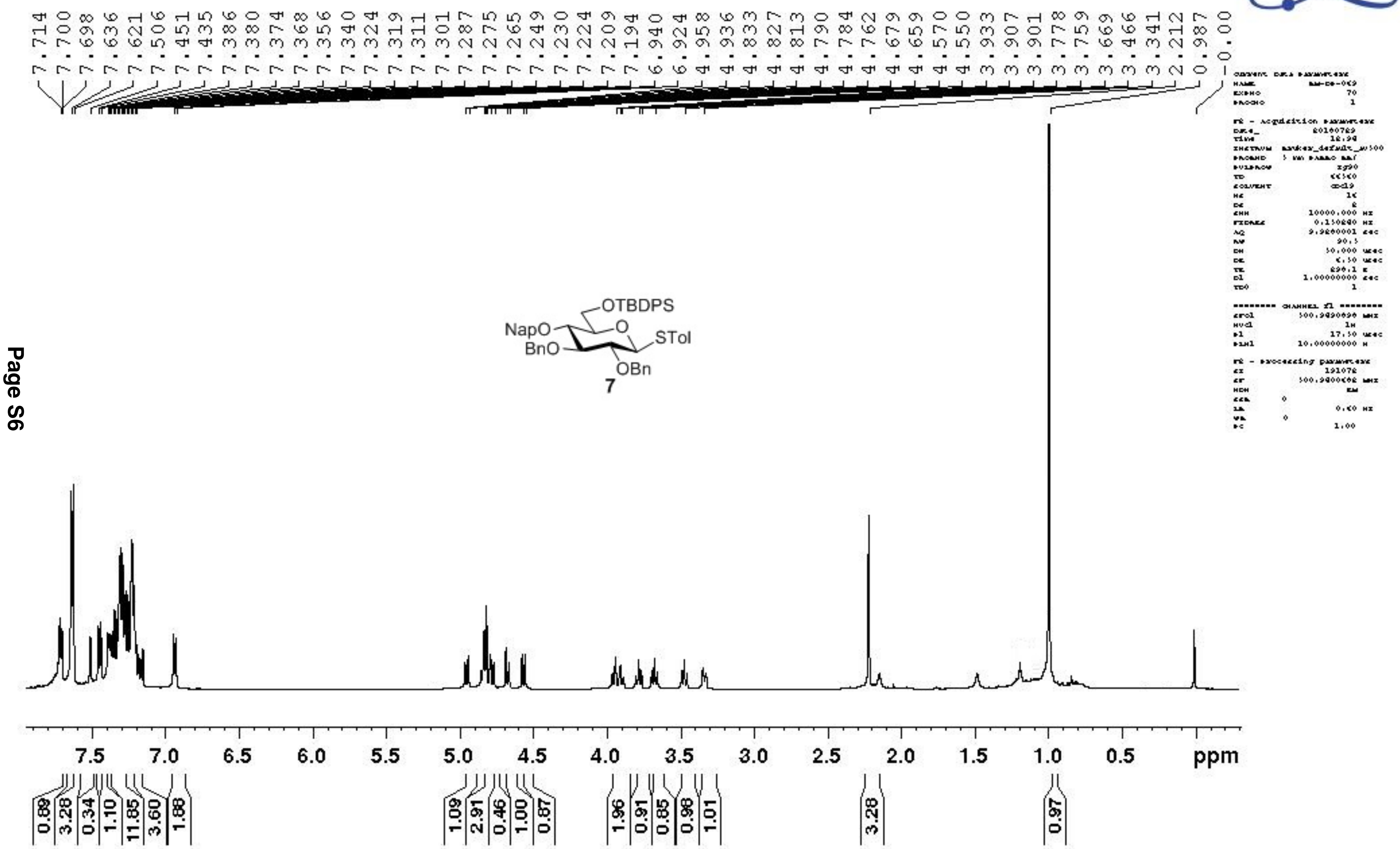

GROUP BM
BM-DP-063 in CDCl3

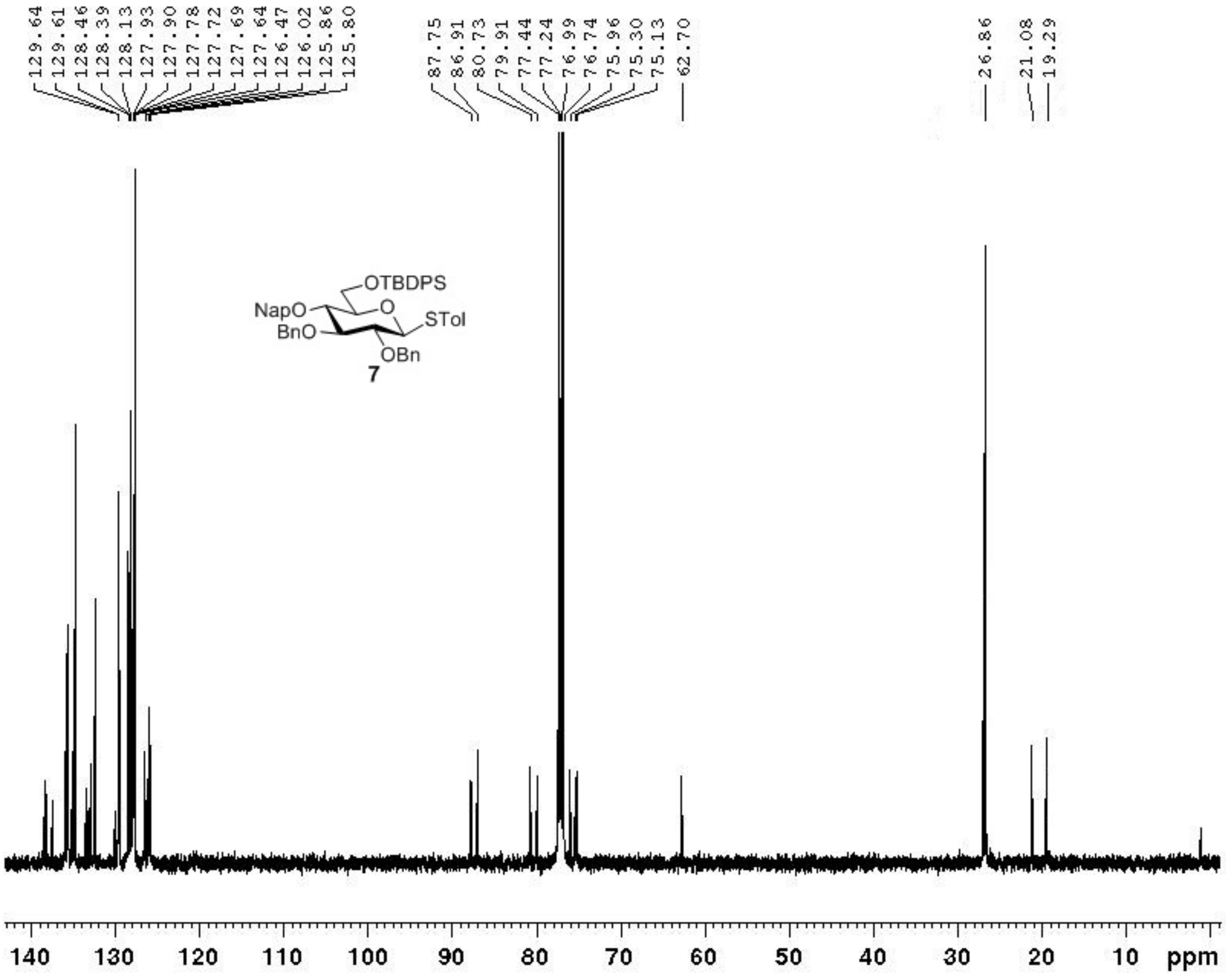


```
Current Data Parameters
NAME             BM-DP-063
EXPNO                   72
PROCNO                   1

F2 - Acquisition Parameters
Date_             20180723
Time                 12.48
INSTRUM  Bruker_default_av50
PROBHD   5 mm PABBO BB/
PULPROG             zgpg30
TD                   65536
SOLVENT              CDCl3
NS                     308
DS                       4
SWH             43859.648 Hz
FIDRES           0.669245 Hz
AQ              0.7471104 sec
RG                     203
DW                  11.400 usec
DE                    6.50 usec
TE                   298.3 K
D1              2.00000000 sec
D11             0.03000000 sec
TD0                      1

-------- CHANNEL f1 --------
SFO1          125.8294646 MHz
NUC1                   13C
P1                   11.50 usec
PLW1           66.00000000 W

-------- CHANNEL f2 --------
SFO2          500.3420014 MHz
NUC2                    1H
CPDPRG[2           waltz16
PCPD2                80.00 usec
PLW2           10.00000000 W
PLW12           0.47852001 W
PLW13           0.24068999 W

F2 - Processing parameters
SI                   32768
SF            125.8106021 MHz
WDW                     EM
SSB      0
LB                    1.00 Hz
GB       0
PC                    1.40
```

GROUP BM
BM-DP-063 in CDCl3

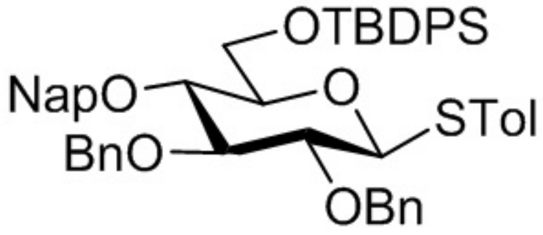

7

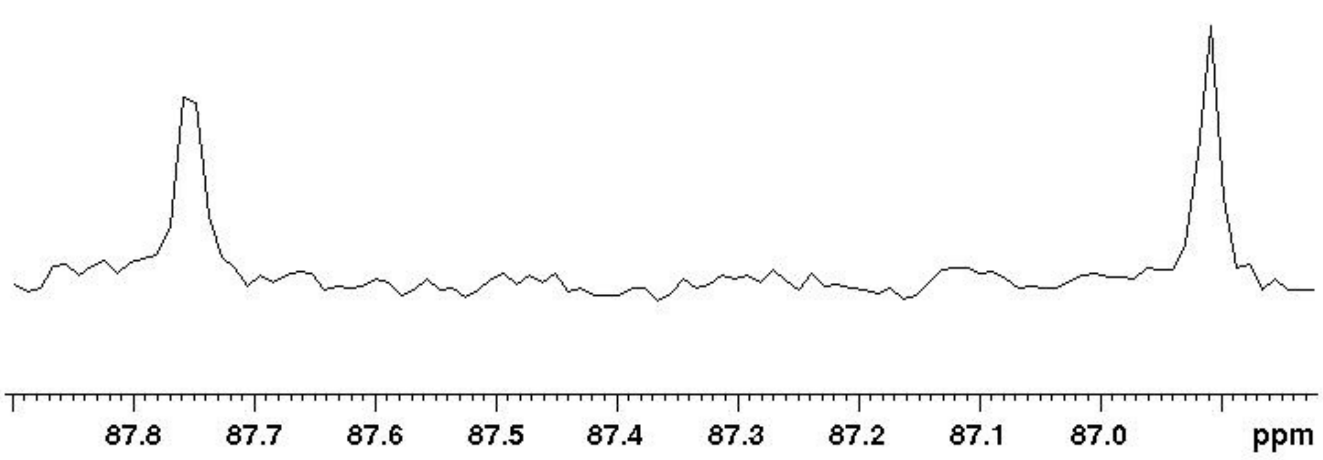


```
Current Data Parameters
NAME          BM-DP-063
EXPNO                72
PROCNO                1

F2 - Acquisition Parameters
Date_          20180723
Time              12.48
INSTRUM  Bruker_default_av50(
PROBHD   5 mm PABBO BB/
PULPROG          zgpg30
TD                65536
SOLVENT           CDCl3
NS                  308
DS                    4
SWH           43859.648 Hz
FIDRES         0.669245 Hz
AQ            0.7471104 sec
RG                  203
DW               11.400 usec
DE                 6.50 usec
TE                298.3 K
D1           2.00000000 sec
D11          0.03000000 sec
TD0                   1

-------- CHANNEL f1 --------
SFO1        125.8294646 MHz
NUC1                13C
P1                11.50 usec
PLW1        66.00000000 W

-------- CHANNEL f2 --------
SFO2        500.3420014 MHz
NUC2                 1H
CPDPRG[2        waltz16
PCPD2             80.00 usec
PLW2        10.00000000 W
PLW12        0.47852001 W
PLW13        0.24068999 W

F2 - Processing parameters
SI                32768
SF          125.8106021 MHz
WDW                  EM
SSB      0
LB                 1.00 Hz
GB       0
PC                 1.40
```

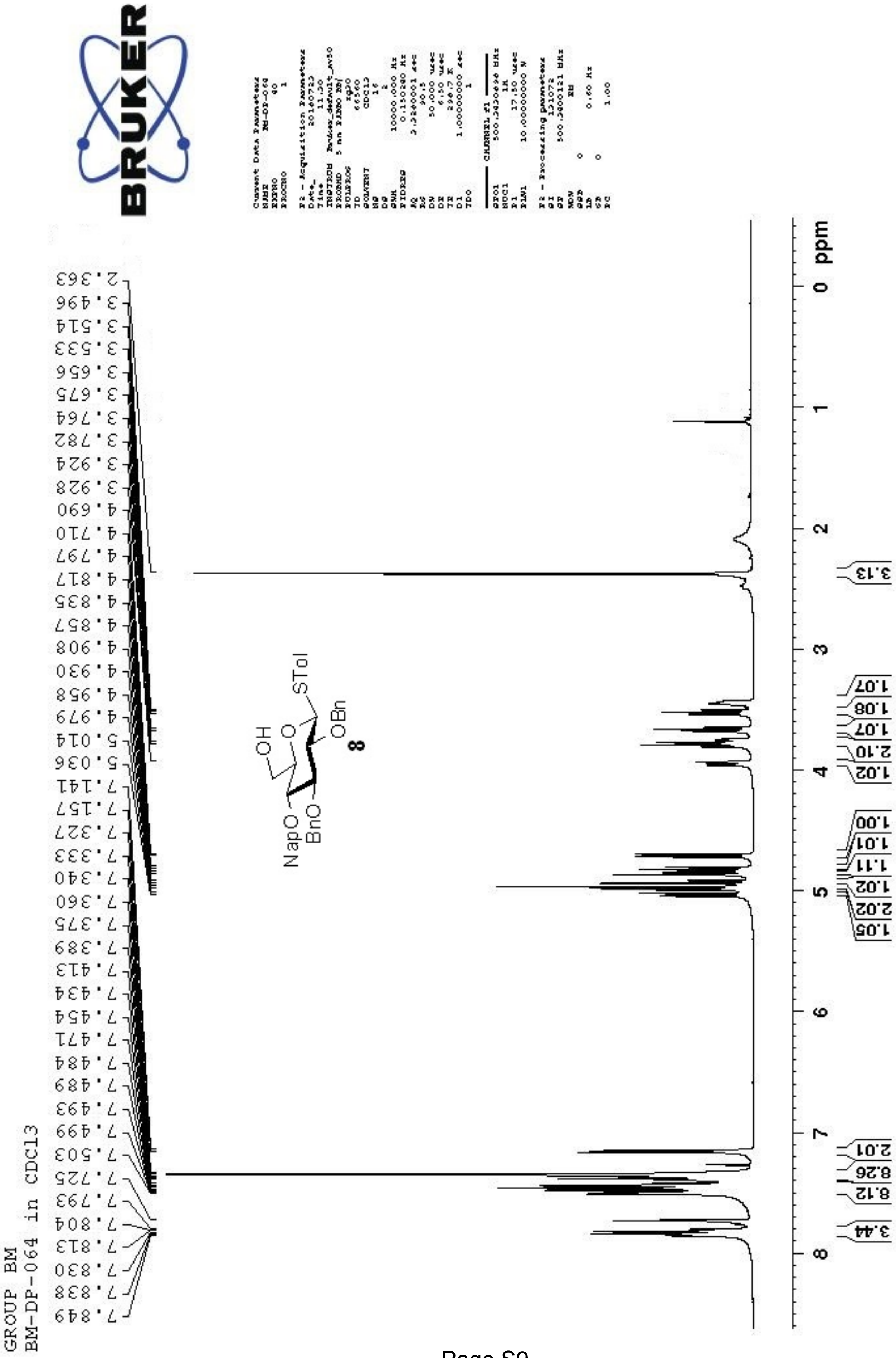
GROUP BM
BM-DP-064 in CDCl3
7.849
7.838
7.830
7.813
7.804
7.793
7.725
7.503
7.499
7.493
7.489
7.484
7.471
7.454
7.434
7.413
7.389
7.375
7.360
7.340
7.333
7.327
7.157
7.141
5.036
5.014
4.979
4.958
4.930
4.908
4.857
4.835
4.817
4.797
4.710
4.690
3.928
3.924
3.782
3.764
3.675
3.656
3.533
3.514
3.496
2.363
BRUKER
NapO
BnO
OH
O
STol
OBn
8
3.44
8.12
8.26
2.01
1.05
2.02
1.02
1.11
1.01
1.00
1.02
2.10
1.07
1.08
1.07
3.13
8
7
6
5
4
3
2
1
0
ppm

GROUP BM
BM-DP-064 in CDCl3

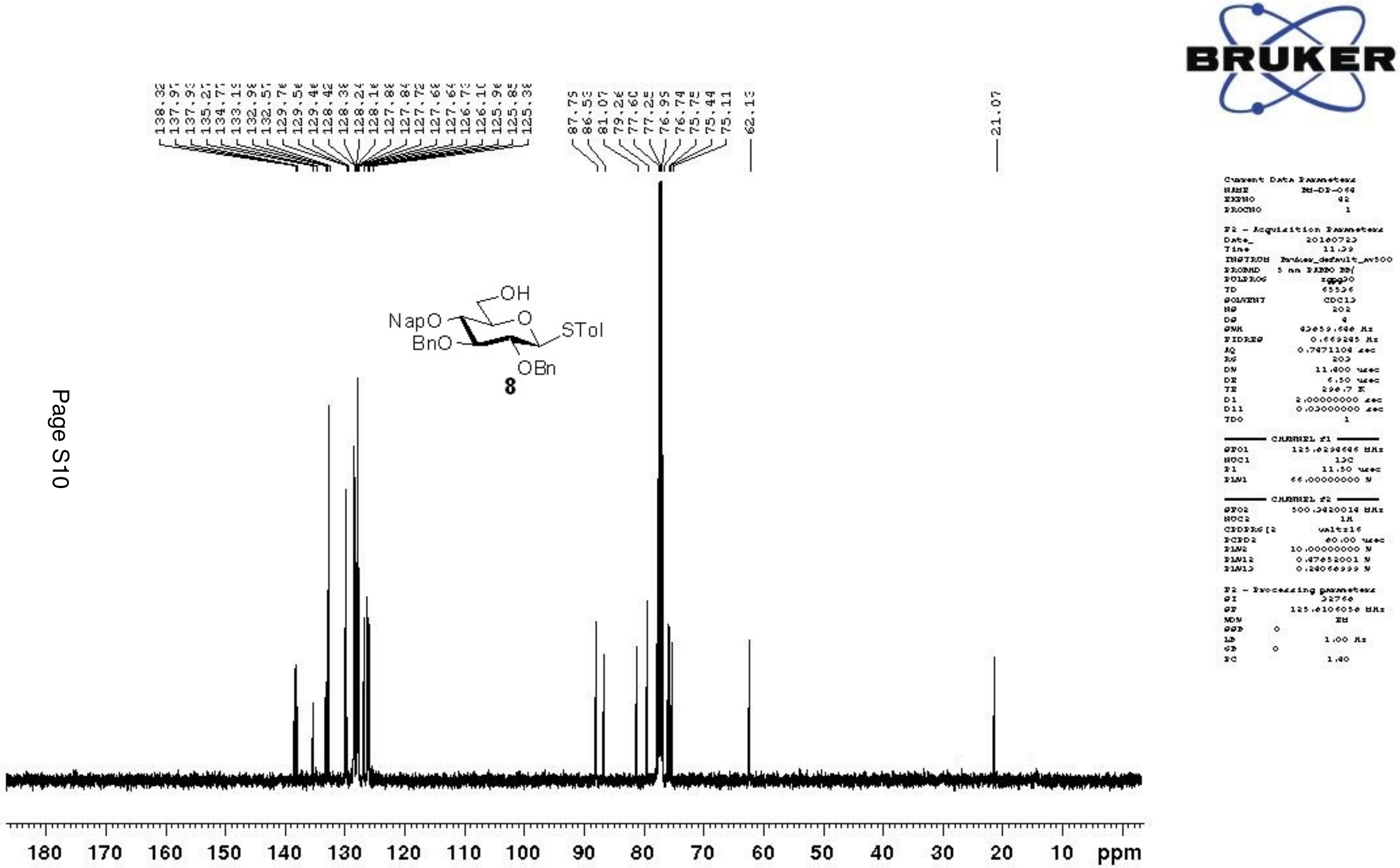

GROUP BM
BM-DP-065 in CDCl3

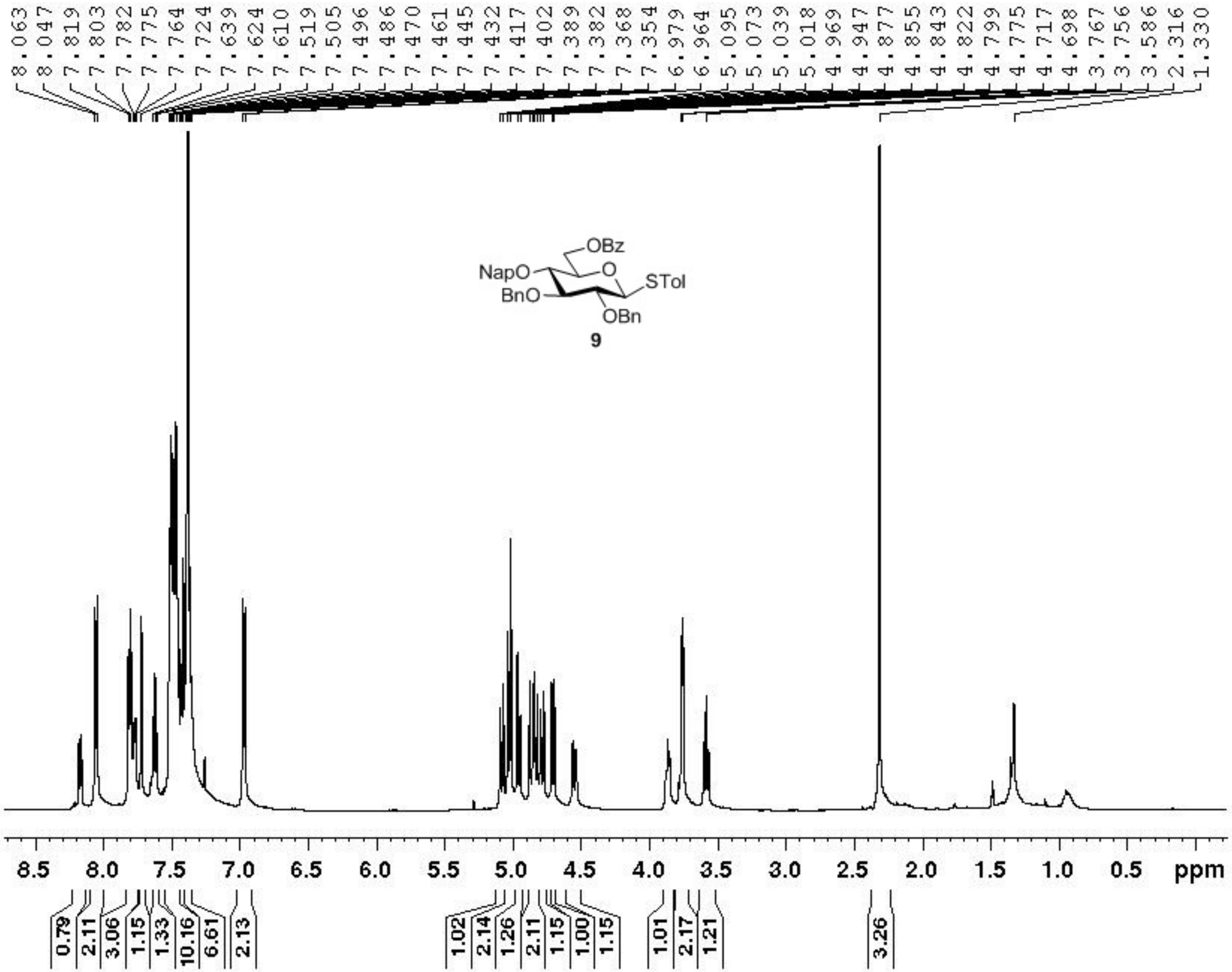

GROUP BM
BM-DP-065 in CDCl3

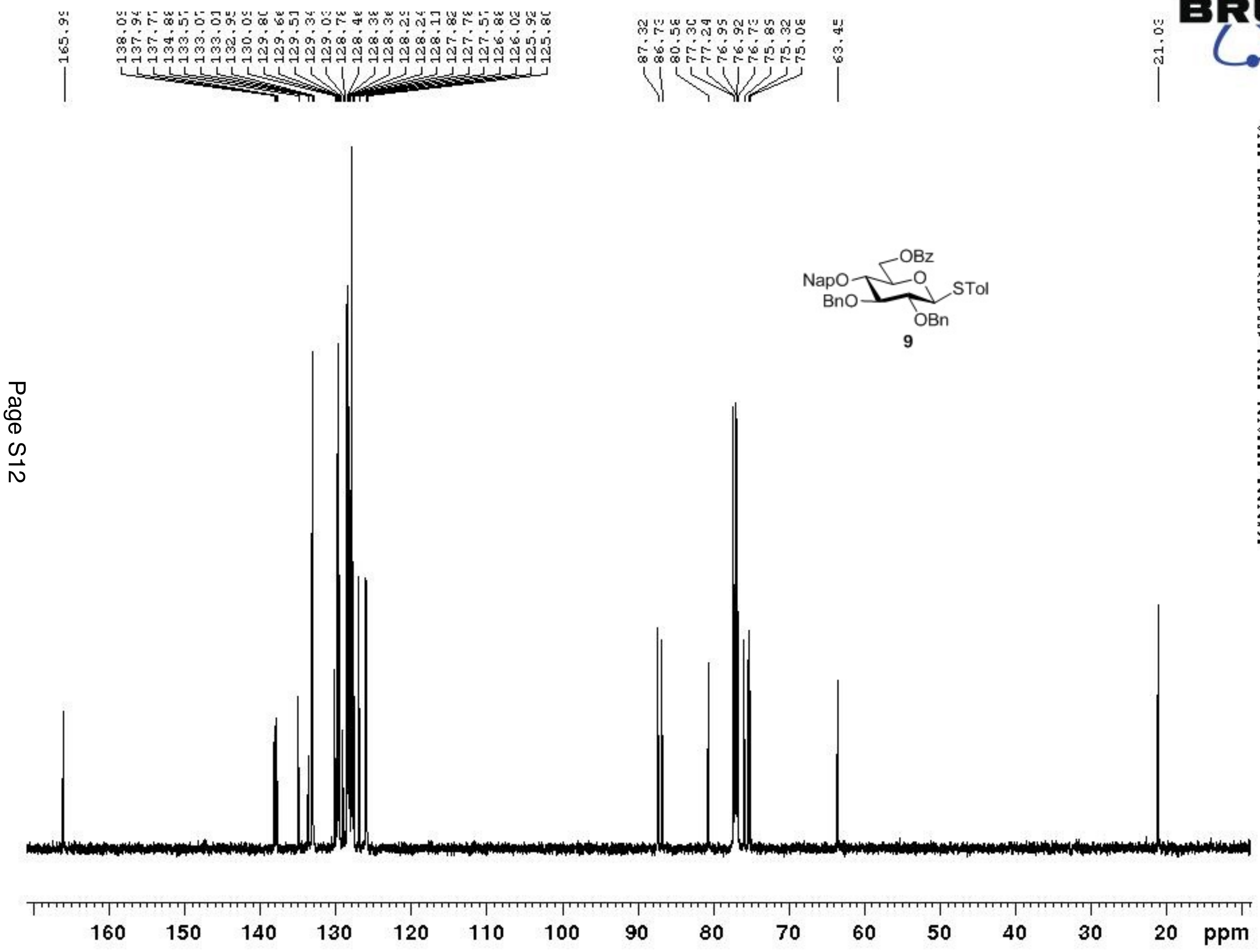

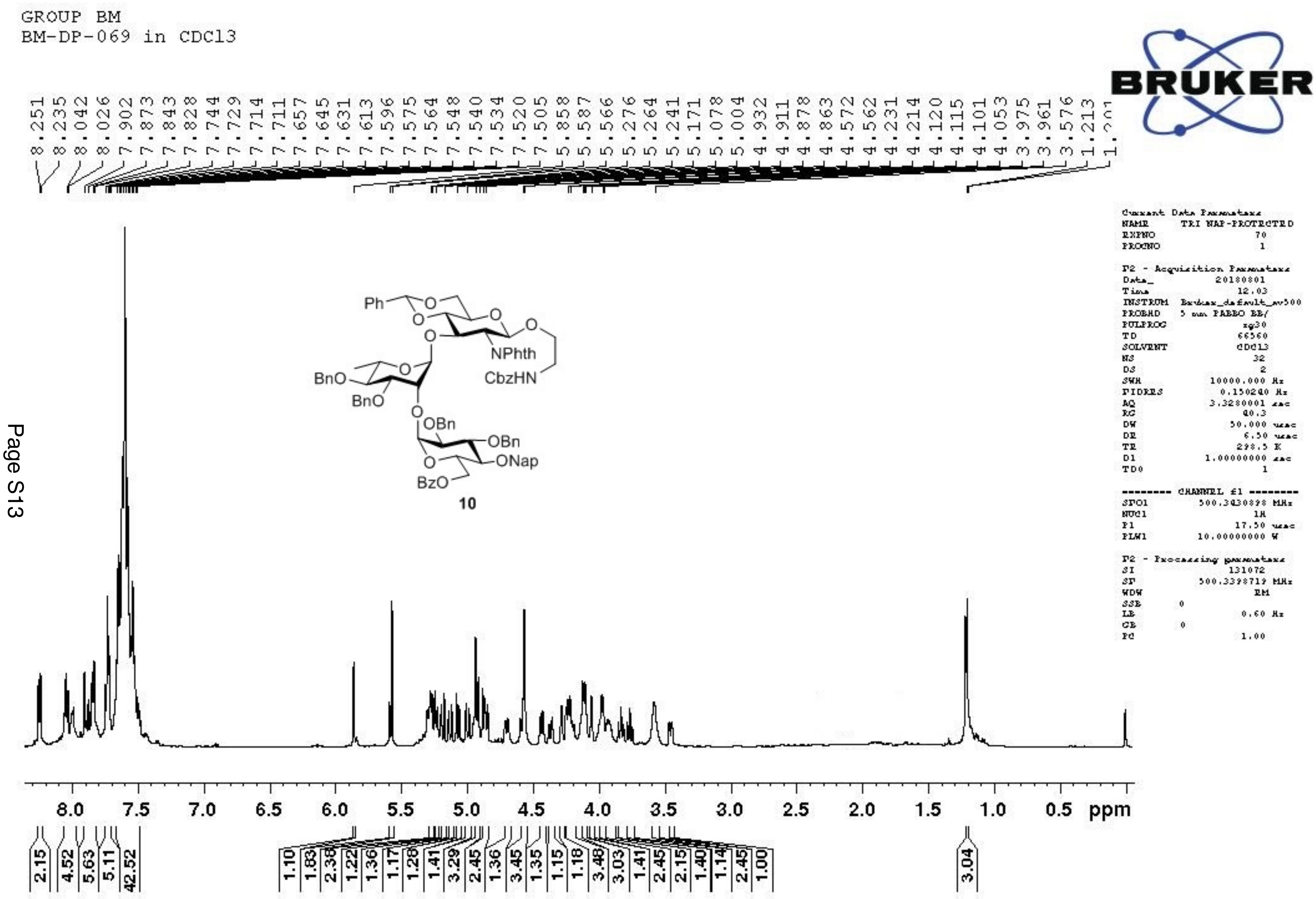
GROUP BM
BM-DP-069 in CDCl3
BRUKER
8.251
8.235
8.042
8.026
7.902
7.873
7.843
7.828
7.744
7.729
7.714
7.711
7.657
7.645
7.631
7.613
7.596
7.575
7.564
7.548
7.540
7.534
7.520
7.505
5.858
5.587
5.566
5.276
5.264
5.241
5.171
5.078
5.004
4.932
4.911
4.878
4.863
4.572
4.562
4.231
4.214
4.120
4.115
4.101
4.053
3.975
3.961
3.576
1.213
Current Data Parameters
NAME TRI NAP-PROTECTED
EXPNO 70
PROCNO 1
F2 - Acquisition Parameters
Date_ 20180801
Time 12.03
INSTRUM Bruker_default_av500
PROBHD 5 mm PABBO BB/
PULPROG zg30
TD 66560
SOLVENT CDCl3
NS 32
DS 2
SWH 10000.000 Hz
FIDRES 0.150240 Hz
AQ 3.3280001 sec
RG 40.3
DW 50.000 usec
DE 6.50 usec
TE 298.5 K
D1 1.00000000 sec
TD0 1
======== CHANNEL f1 ========
SFO1 500.3430898 MHz
NUC1 1H
P1 17.50 usec
PLW1 10.00000000 W
F2 - Processing parameters
SI 131072
SF 500.3398719 MHz
WDW EM
SSB 0
LB 0.60 Hz
GB 0
PC 1.00
Ph
O
O
O
O
NPhth
CbzHN
BnO
BnO
O
OBn
OBn
ONap
BzO
10
8.0
7.5
7.0
6.5
6.0
5.5
5.0
4.5
4.0
3.5
3.0
2.5
2.0
1.5
1.0
0.5
ppm
2.15
4.52
5.63
5.11
42.52
1.10
1.83
2.38
1.22
1.36
1.17
1.28
1.41
3.29
2.45
1.36
3.45
1.35
1.15
1.18
3.48
3.03
1.41
2.45
2.15
1.40
1.14
2.45
1.00
3.04

GROUP BM
BM-DP-069 in CDCl3

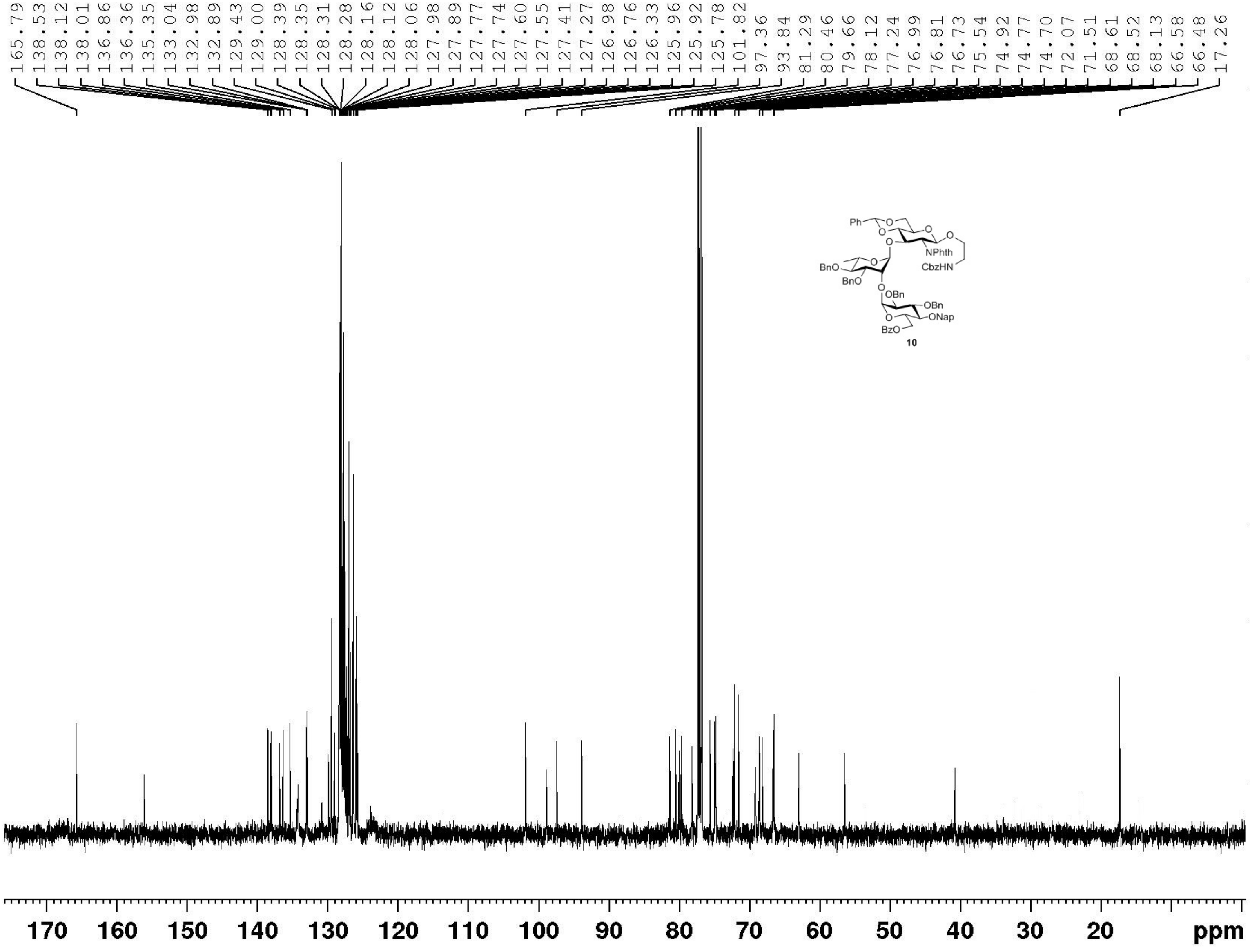

10

Current Data Parameters
NAME TRI NAP-PROTECTED
EXPNO 71
PROCNO 1

F2 - Acquisition Parameters
Date_ 20180801
Time 12.09
INSTRUM Bruker_default_av500
PROBHD 5 mm PABBO BB/
PULPROG zgpg30
TD 65536
SOLVENT CDCl3
NS 199
DS 4
SWH 43859.648 Hz
FIDRES 0.669245 Hz
AQ 0.7471104 sec
RG 203
DW 11.400 usec
DE 6.50 usec
TE 299.2 K
D1 2.00000000 sec
D11 0.03000000 sec
TD0 1

======== CHANNEL f1 ========
SFO1 125.8294646 MHz
NUC1 13C
P1 11.50 usec
PLW1 66.00000000 W

======== CHANNEL f2 ========
SFO2 500.3420014 MHz
NUC2 1H
CPDPRG[2 waltz16
PCPD2 80.00 usec
PLW2 10.00000000 W
PLW12 0.47852001 W
PLW13 0.24068999 W

F2 - Processing parameters
SI 32768
SF 125.8106095 MHz
WDW EM
SSB 0
LB 1.00 Hz
GB 0
PC 1.40

GROUP BM
BM-DP-082 in CDCl3

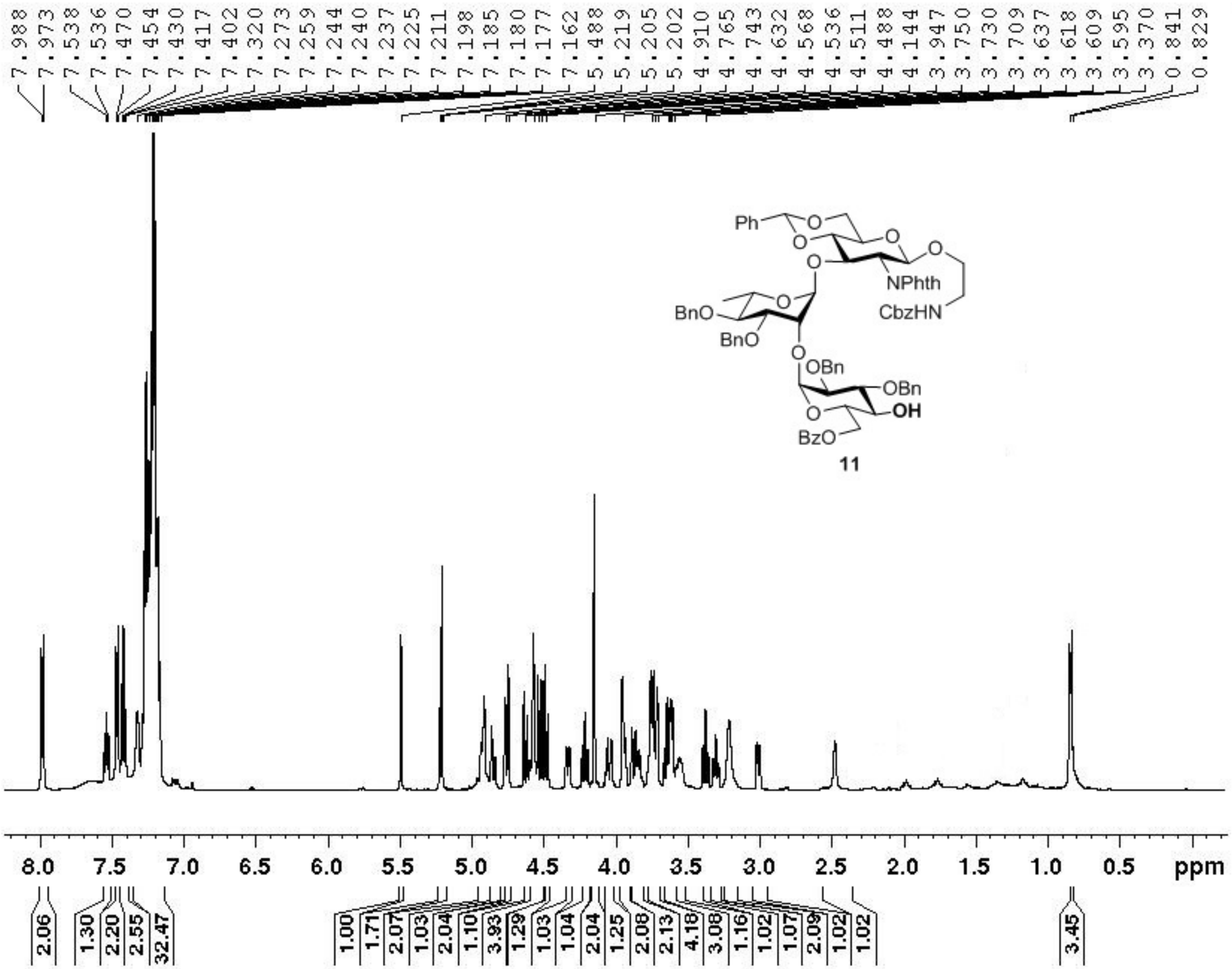

BRUKER

Current Data Parameters
NAME TRI ACCEPTOR
EXPNO 10
PROCNO 1

F2 - Acquisition Parameters
Date_ 20181001
Time 11.11
INSTRUM Bruker_default_av500
PROBHD 5 mm PABBO BB/
PULPROG zg30
TD 66560
SOLVENT CDCl3
NS 16
DS 2
SWH 10000.000 Hz
FIDRES 0.150240 Hz
AQ 3.3280001 sec
RG 50.8
DW 50.000 usec
DE 6.50 usec
TE 2261.6 K
D1 1.00000000 sec
TD0 1

CHANNEL f1
SFO1 500.3430898 MHz
NUC1 1H
P1 17.50 usec
PLW1 10.00000000 W

F2 - Processing parameters
SI 131072
SF 500.3400527 MHz
WDW EM
SSB 0
LB 0.30 Hz
GB 0
PC 1.00

GROUP BM
BM-DP-082 in CDCl3

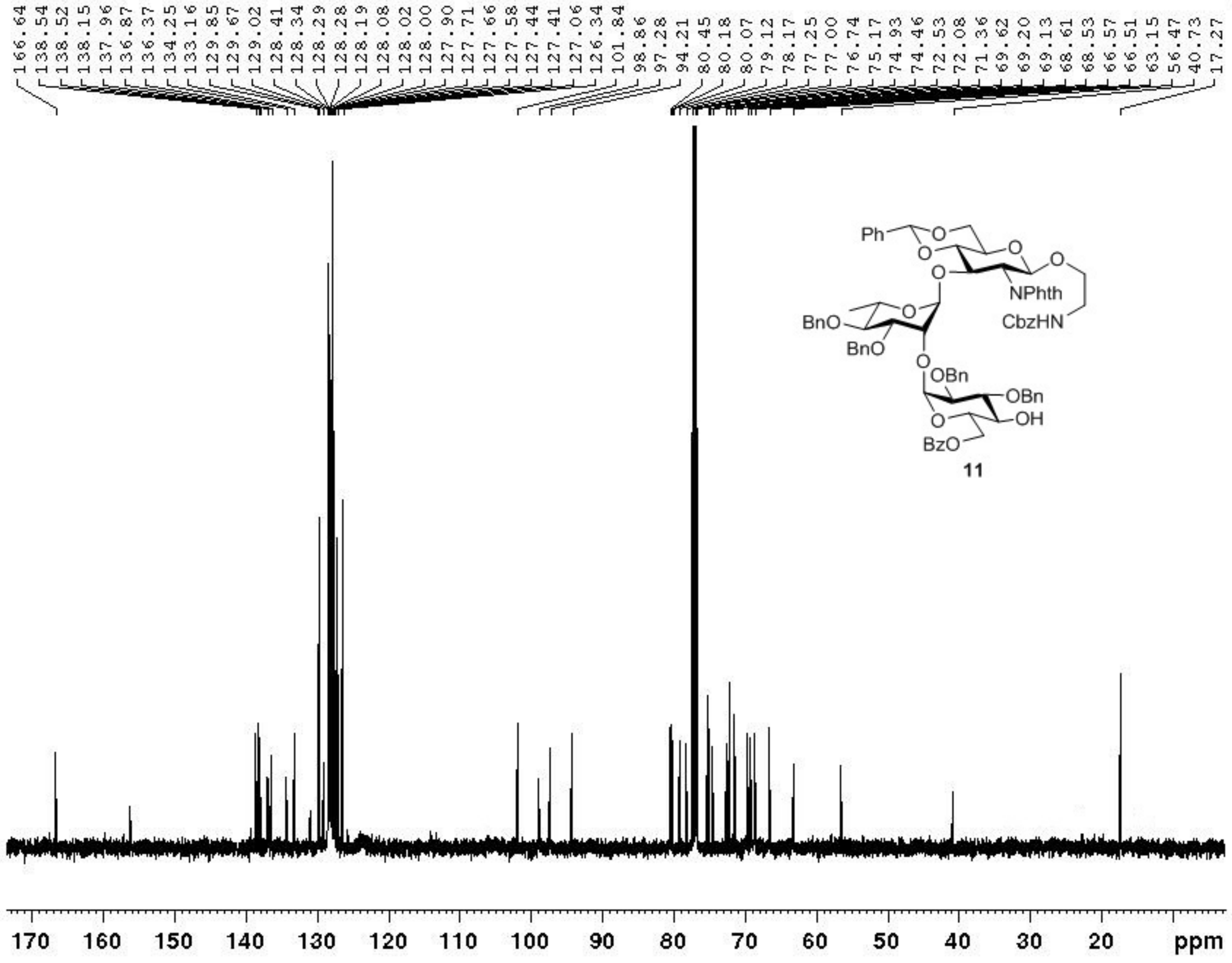

11

Current Data Parameters
NAME TRI ACCEPTOR
EXPNO 13
PROCNO 1

F2 - Acquisition Parameters
Date_ 20181001
Time 11.39
INSTRUM Bruker_default_av50
PROBHD 5 mm PABBO BB/
PULPROG zgpg30
TD 65536
SOLVENT CDCl3
NS 156
DS 4
SWH 43859.648 Hz
FIDRES 0.669245 Hz
AQ 0.7471104 sec
RG 203
DW 11.400 usec
DE 6.50 usec
TE 2260.0 K
D1 2.00000000 sec
D11 0.03000000 sec
TD0 1

======== CHANNEL f1 ========
SFO1 125.8294646 MHz
NUC1 13C
P1 11.50 usec
PLW1 66.00000000 W

======== CHANNEL f2 ========
SFO2 500.3420014 MHz
NUC2 1H
CPDPRG[2 waltz16
PCPD2 80.00 usec
PLW2 10.00000000 W
PLW12 0.47852001 W
PLW13 0.24068999 W

F2 - Processing parameters
SI 32768
SF 125.8106070 MHz
WDW EM
SSB 0
LB 1.00 Hz
GB 0
PC 1.10

GROUP BM
BM-DP-076 in CDCl3

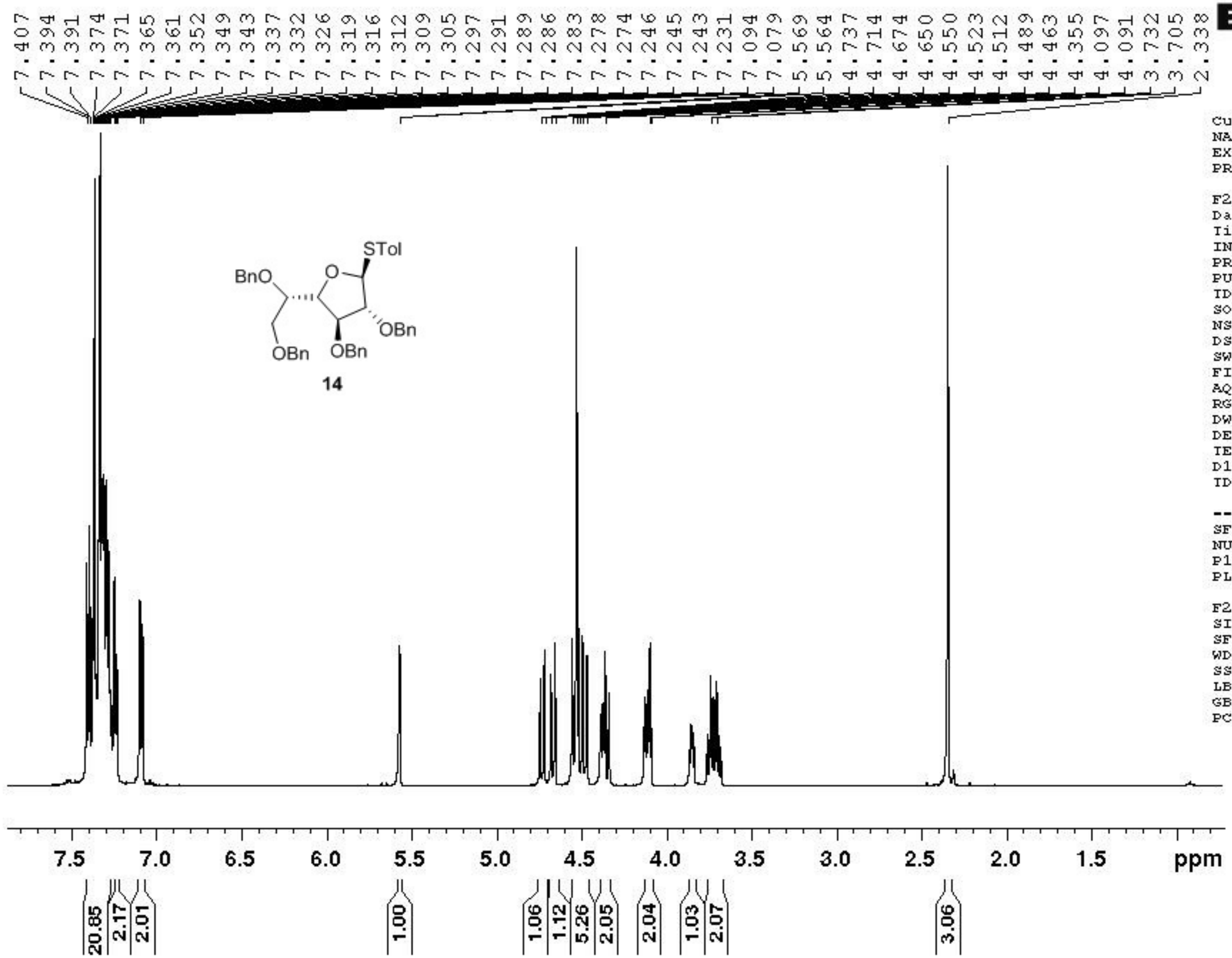


```
Current Data Parameters
NAME          BM-DP-076
EXPNO               230
PROCNO                1

F2 - Acquisition Parameters
Date_          20180927
Time              16.35
INSTRUM  Bruker_default_av5(
PROBHD   5 mm PABBO BB/
PULPROG            zg30
TD                66560
SOLVENT           CDCl3
NS                   16
DS                    2
SWH           10000.000 Hz
FIDRES         0.150240 Hz
AQ            3.3280001 sec
RG                 45.2
DW               50.000 use(
DE                 6.50 use(
TE               2237.4 K
D1           1.00000000 sec
TD0                   1

-------- CHANNEL f1 --------
SFO1        500.3430898 MHz
NUC1                 1H
P1                17.50 use(
PLW1        10.00000000 W

F2 - Processing parameters
SI               131072
SF          500.3400186 MHz
WDW                  EM
SSB      0
LB                 0.30 Hz
GB       0
PC                 1.00
```

GROUP BM
BM-DP-076 in CDCl3

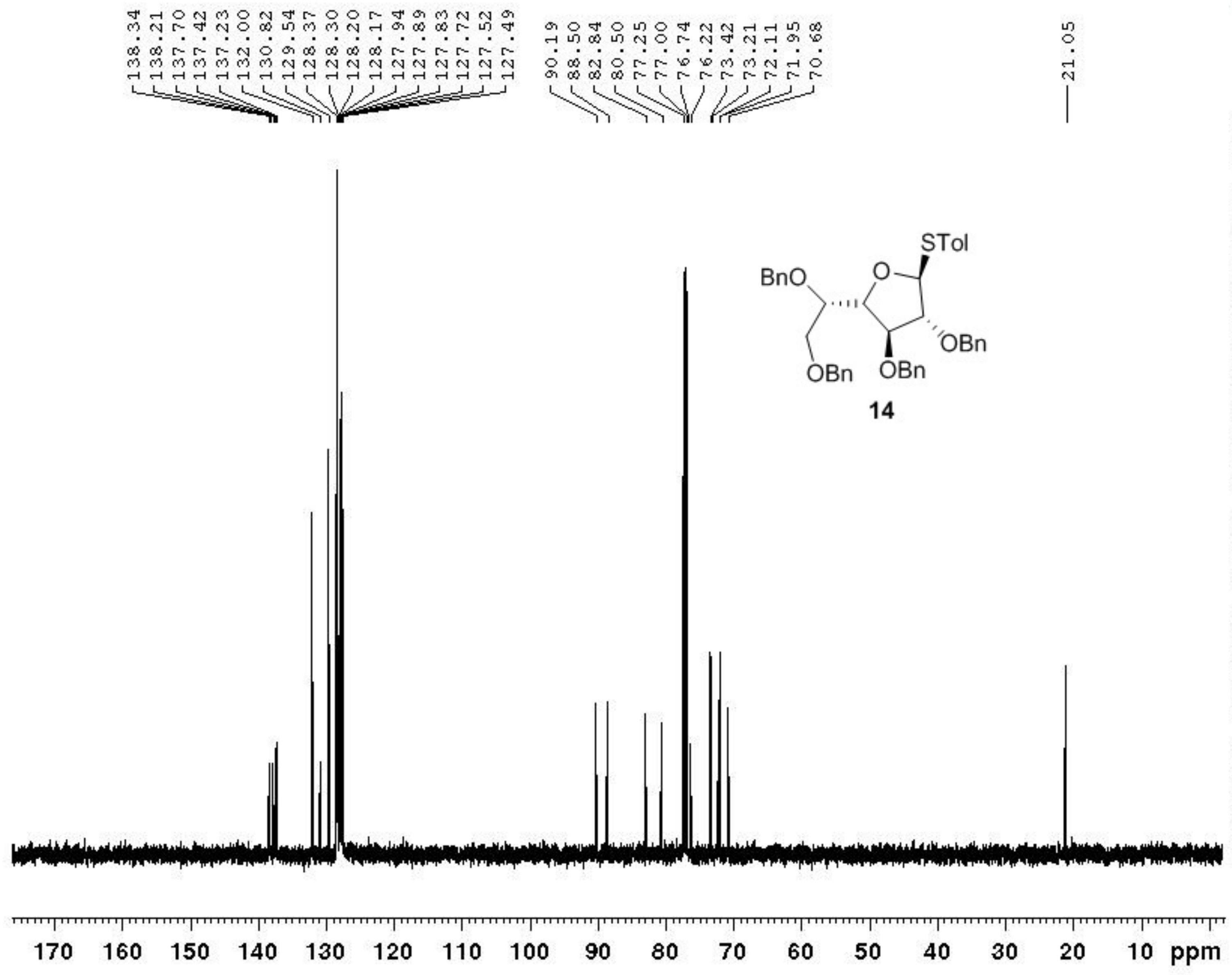


```
Current Data Parameters
NAME              BM-DP-076
EXPNO                   231
PROCNO                    1

F2 - Acquisition Parameters
Date_              20180927
Time                  16.41
INSTRUM  Bruker_default_av50(
PROBHD   5 mm PABBO BB/
PULPROG             zgpg30
TD                   65536
SOLVENT              CDCl3
NS                      51
DS                       4
SWH             43859.648 Hz
FIDRES           0.669245 Hz
AQ              0.7471104 sec
RG                     203
DW                  11.400 usec
DE                    6.50 usec
TE                  2250.9 K
D1              2.00000000 sec
D11             0.03000000 sec
TD0                      1

-------- CHANNEL f1 --------
SFO1          125.8294646 MHz
NUC1                   13C
P1                   11.50 usec
PLW1           66.00000000 W

-------- CHANNEL f2 --------
SFO2          500.3420014 MHz
NUC2                    1H
CPDPRG[2            waltz16
PCPD2                80.00 usec
PLW2           10.00000000 W
PLW12           0.47852001 W
PLW13           0.24068999 W

F2 - Processing parameters
SI                   32768
SF            125.8106059 MHz
WDW                     EM
SSB      0
LB                    1.00 Hz
GB       0
PC                    1.10
```

GROUP BM
BM-DP-077ALPHA in CDCl3

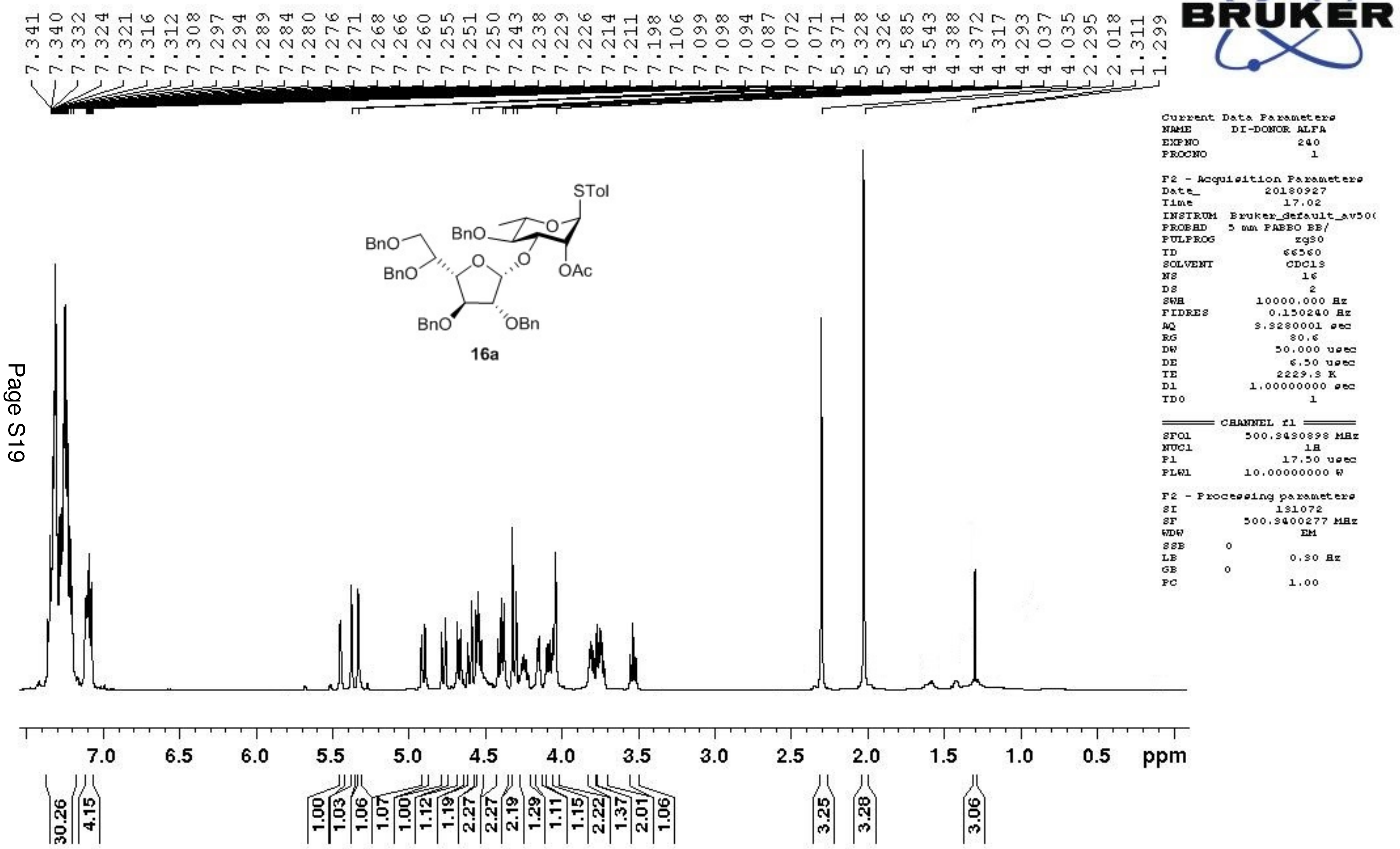

GROUP BM
BM-DP-077ALPHA in CDCl3

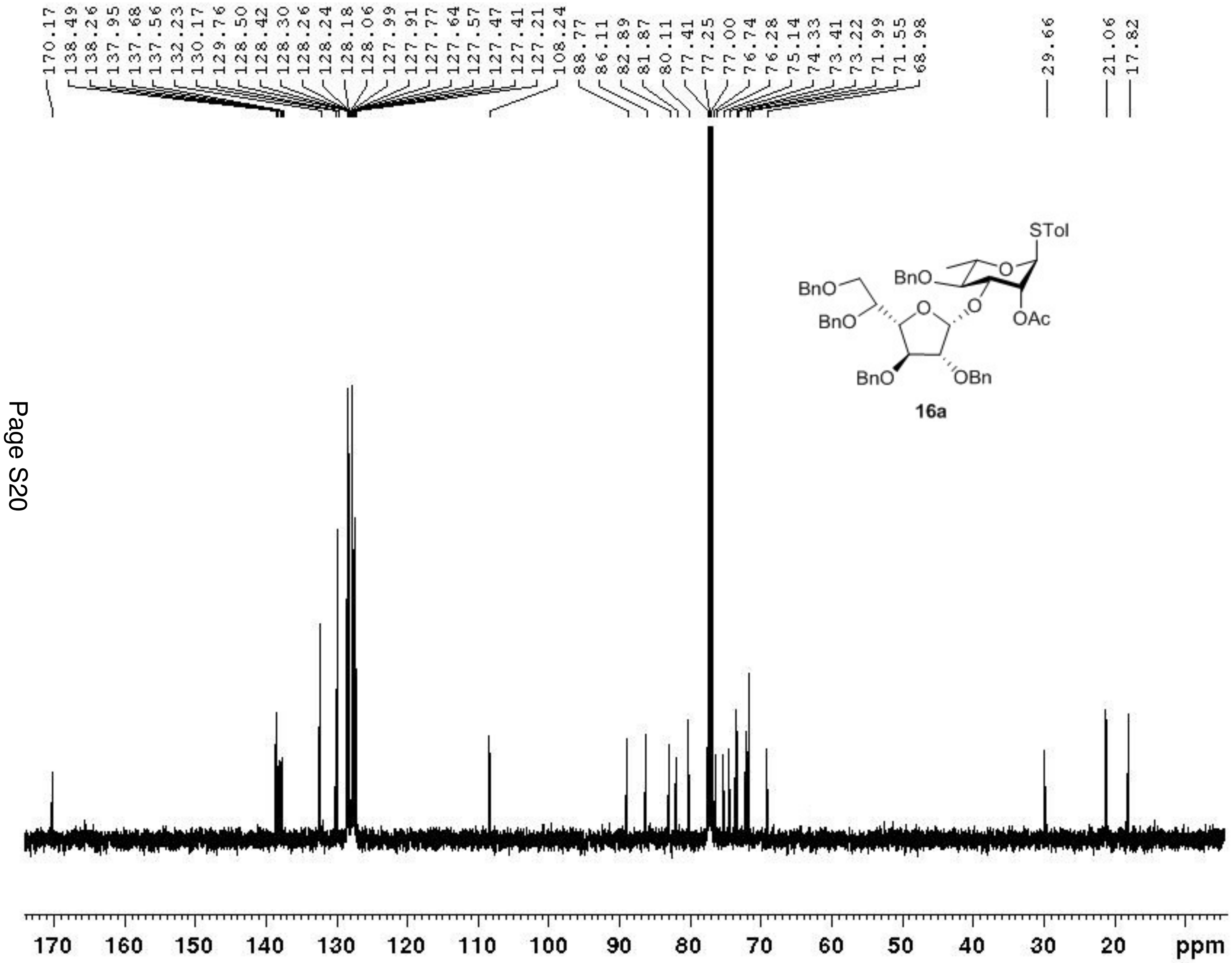

16a

```
Current Data Parameters
NAME          DI-DONOR ALFA
EXPNO                   241
PROCNO                    1

F2 - Acquisition Parameters
Date_              20180927
Time                  17.05
INSTRUM   Bruker_default_av500
PROBHD     5 mm PABBO BB/
PULPROG             zgpg30
TD                   65536
SOLVENT              CDCl3
NS                     136
DS                       4
SWH             43859.648 Hz
FIDRES           0.669245 Hz
AQ              0.7471104 sec
RG                     203
DW                  11.400 usec
DE                    6.50 usec
TE                  2260.0 K
D1             2.00000000 sec
D11            0.03000000 sec
TD0                      1

-------- CHANNEL f1 --------
SFO1          125.8294646 MHz
NUC1                   13C
P1                   11.50 usec
PLW1          66.00000000 W

-------- CHANNEL f2 --------
SFO2          500.3420014 MHz
NUC2                    1H
CPDPRG[2           waltz16
PCPD2                80.00 usec
PLW2          10.00000000 W
PLW12          0.47852001 W
PLW13          0.24068999 W

F2 - Processing parameters
SI                   32768
SF            125.8106004 MHz
WDW                     EM
SSB       0
LB                    1.00 Hz
GB        0
PC                    1.10
```

GROUP BM
BM-DP-077BETA in CDCl3

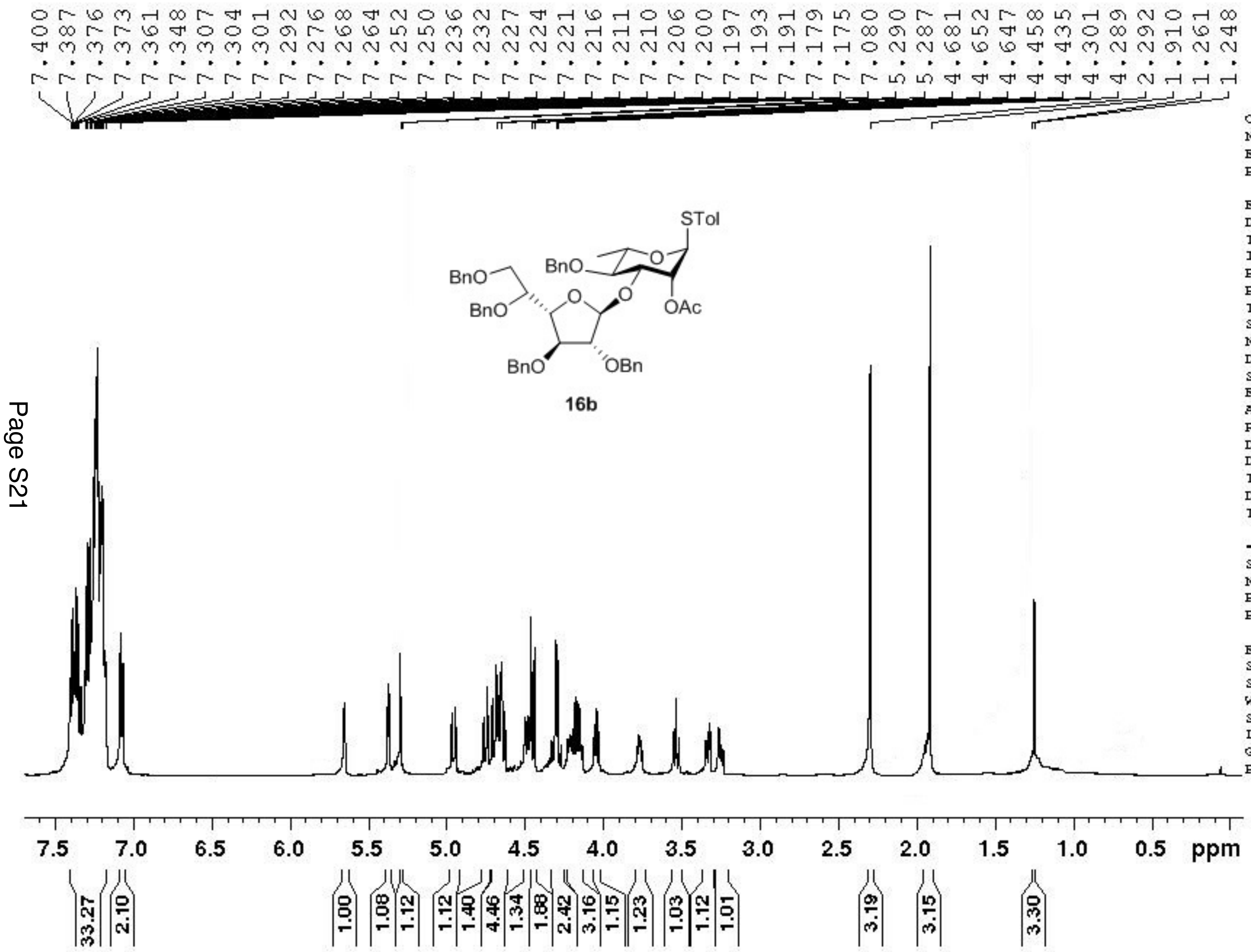

Current Data Parameters		
NAME	DI-DONOR BETA	
EXPNO	250	
PROCNO	1	
F2 - Acquisition Parameters		
Date_	20180927	
Time	17.31	
INSTRUM	Bruker_default_av500	
PROBHD	5 mm PABBO BB/	
PULPROG	zg30	
TD	66560	
SOLVENT	CDCl3	
NS	16	
DS	2	
SWH	10000.000	Hz
FIDRES	0.150240	Hz
AQ	3.3280001	sec
RG	80.6	
DW	50.000	usec
DE	6.50	usec
TE	2238.3	K
D1	1.00000000	sec
TD0	1	
-------- CHANNEL f1 --------		
SFO1	500.3430898	MHz
NUC1	1H	
P1	17.50	usec
PLW1	10.00000000	W
F2 - Processing parameters		
SI	131072	
SF	500.3400277	MHz
WDW	EM	
SSB	0	
LB	0.30	Hz
GB	0	
PC	1.00	

GROUP BM
BM-DP-077BETA in CDCl3

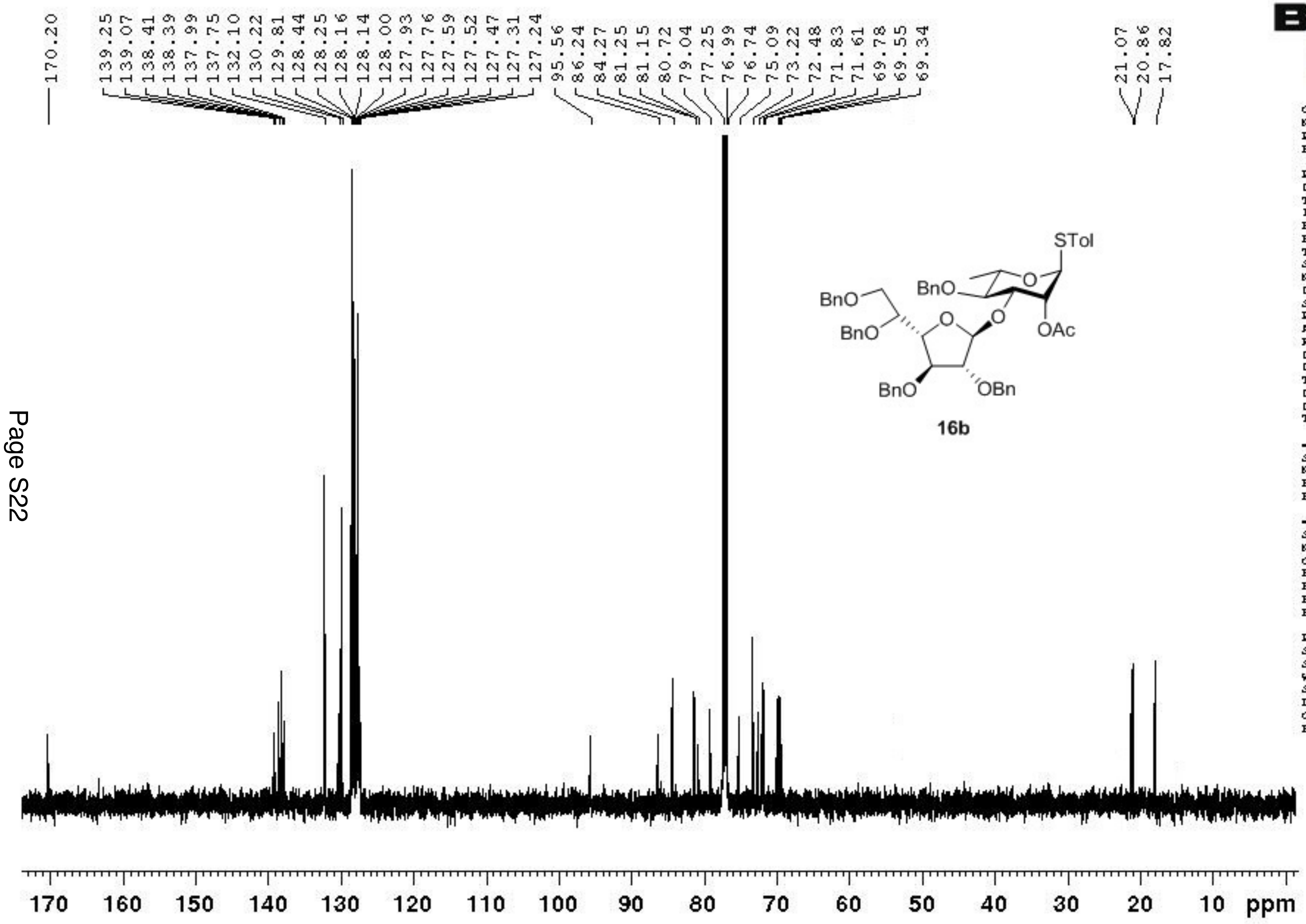

16b

Current Data Parameters
NAME DI-DONOR BETA
EXPNO 251
PROCNO 1

F2 - Acquisition Parameters
Date_ 20180927
Time 17.35
INSTRUM Bruker_default_av500
PROBHD 5 mm PABBO BB/
PULPROG zgpg30
TD 65536
SOLVENT CDCl3
NS 368
DS 4
SWH 43859.648 Hz
FIDRES 0.669245 Hz
AQ 0.7471104 sec
RG 203
DW 11.400 usec
DE 6.50 usec
TE 2277.6 K
D1 2.00000000 sec
D11 0.03000000 sec
TD0 1

======== CHANNEL f1 ========
SFO1 125.8294646 MHz
NUC1 13C
P1 11.50 usec
PLW1 66.00000000 W

======== CHANNEL f2 ========
SFO2 500.3420014 MHz
NUC2 1H
CPDPRG[2 waltz16
PCPD2 80.00 usec
PLW2 10.00000000 W
PLW12 0.47852001 W
PLW13 0.24068999 W

F2 - Processing parameters
SI 32768
SF 125.8105979 MHz
WDW EM
SSB 0
LB 1.00 Hz
GB 0
PC 1.10

GROUP BM
BM-DP-079PENTA in CDCl3

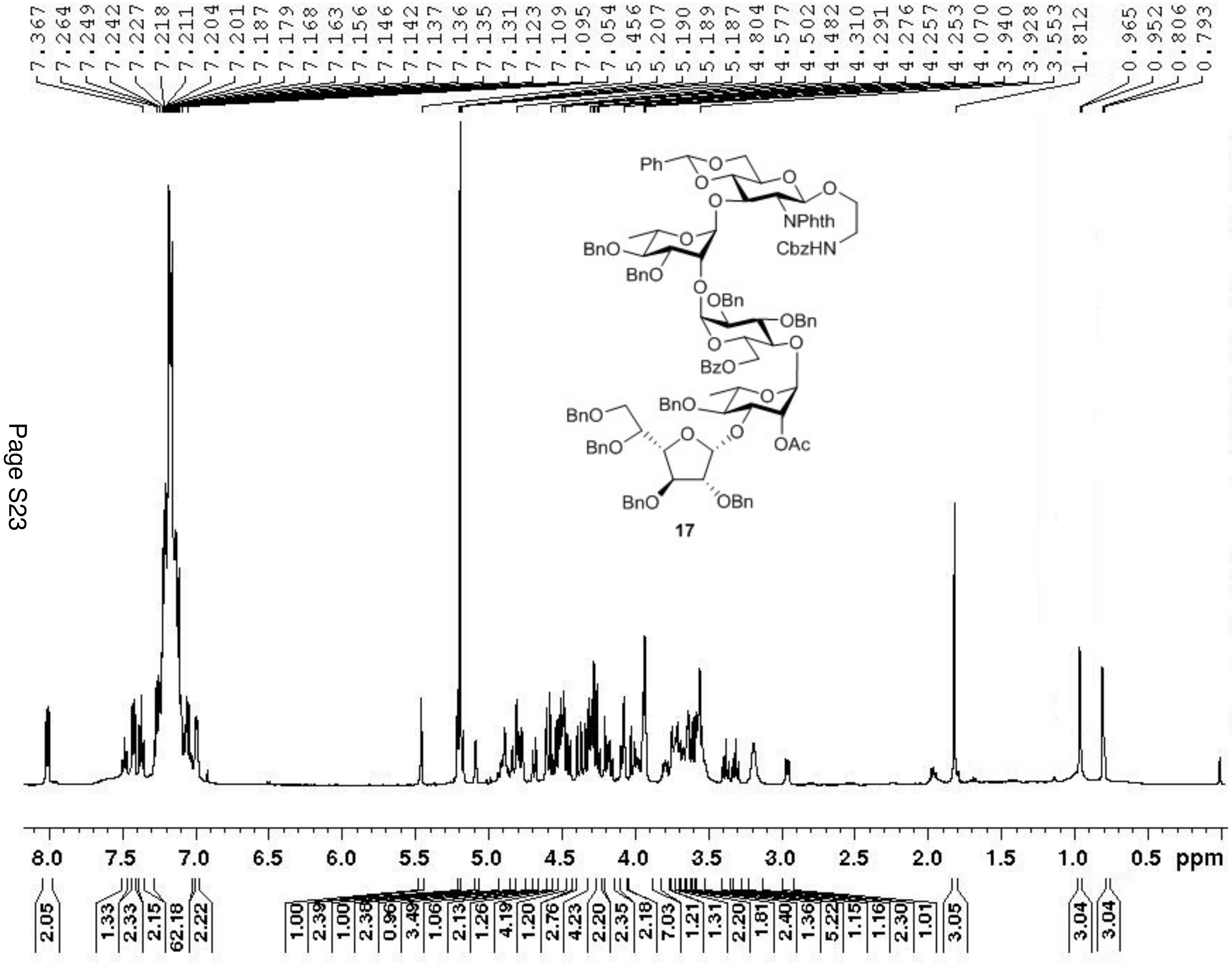

BRUKER

Current Data Parameters
NAME PENTA PROTECTED
EXPNO 290
PROCNO 1

F2 - Acquisition Parameters
Date_ 20180927
Time 18.43
INSTRUM Bruker_default_av5(
PROBHD 5 mm PABBO BB/
PULPROG zg30
TD 66560
SOLVENT CDCl3
NS 16
DS 2
SWH 10000.000 Hz
FIDRES 0.150240 Hz
AQ 3.3280001 sec
RG 50.8
DW 50.000 use(
DE 6.50 use(
TE 2286.4 K
D1 1.00000000 sec
TD0 1

-------- CHANNEL f1 --------
SFO1 500.3430898 MHz
NUC1 1H
P1 17.50 use(
PLW1 10.00000000 W

F2 - Processing parameters
SI 131072
SF 500.3400632 MHz
WDW EM
SSB 0
LB 0.30 Hz
GB 0
PC 1.00

GROUP BM
BM-DP-079PENTA in CDCl3

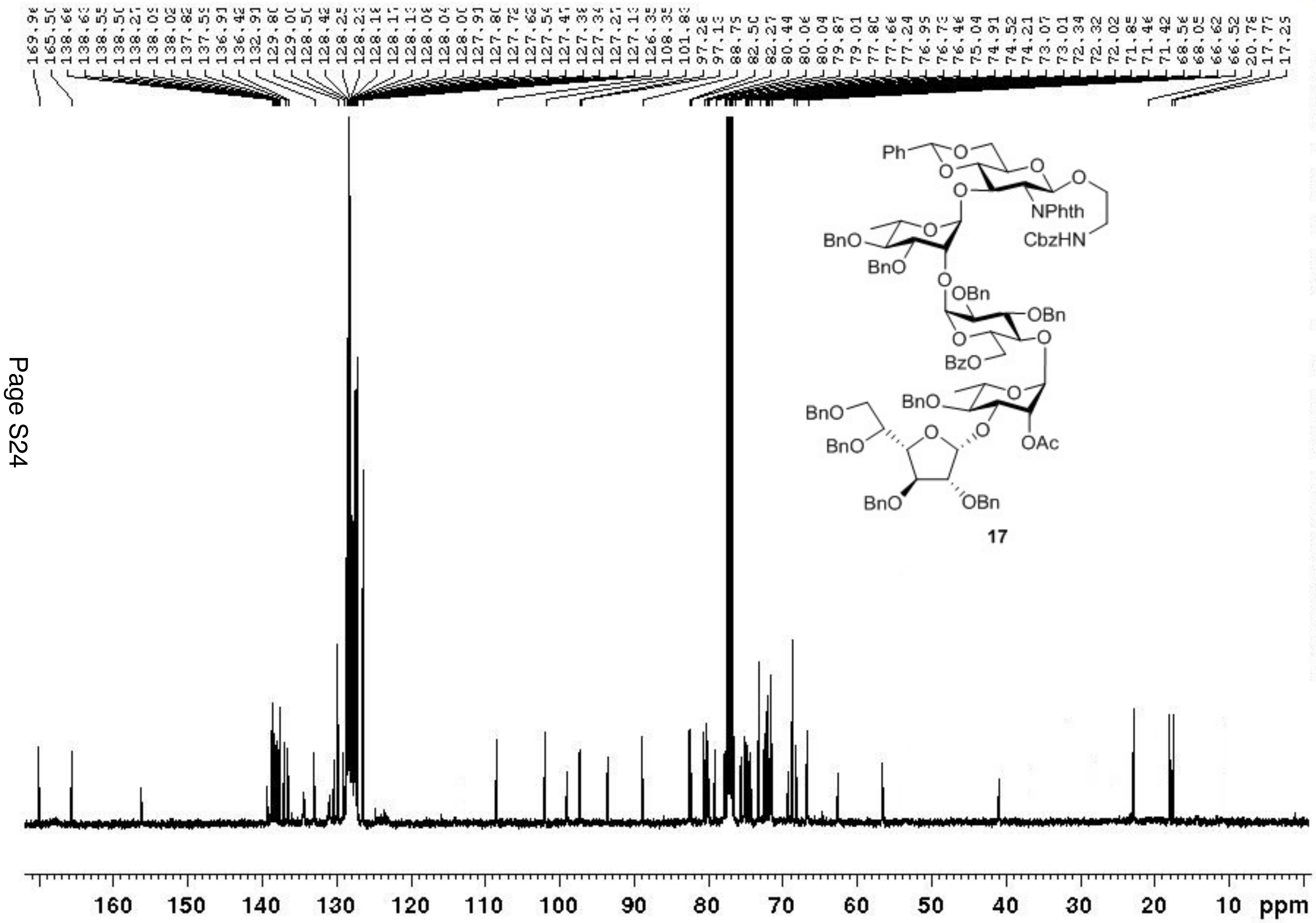

17

Current Data Parameters
NAME PENTA PROTECTED
EXPNO 293
PROCNO 1

F2 - Acquisition Parameters
Date_ 20180927
Time 19.10
INSTRUM Bruker_default_av500
PROBHD 5 mm PABBO BB/
PULPROG zgpg30
TD 65536
SOLVENT CDCl3
NS 5000
DS 4
SWH 43859.648 Hz
FIDRES 0.669245 Hz
AQ 0.7471104 sec
RG 203
DW 11.400 usec
DE 6.50 usec
TE 2259.4 K
D1 2.00000000 sec
D11 0.03000000 sec
TD0 1

======== CHANNEL f1 ========
SFO1 125.8290606 MHz
NUC1 13C
P1 11.50 usec
PLW1 66.00000000 W

======== CHANNEL f2 ========
SFO2 500.3420014 MHz
NUC2 1H
CPDPRG[2 waltz16
PCPD2 80.00 usec
PLW2 10.00000000 W
PLW12 0.47852001 W
PLW13 0.24068999 W

F2 - Processing parameters
SI 32768
SF 125.8106026 MHz
WDW EM
SSB 0
LB 1.00 Hz
GB 0
PC 1.10

GROUP BM

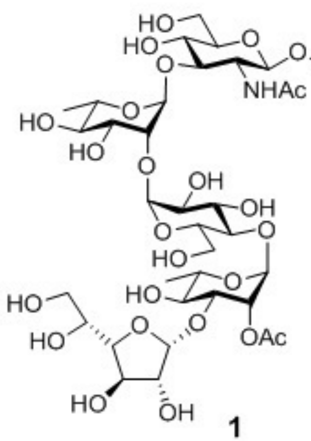

1

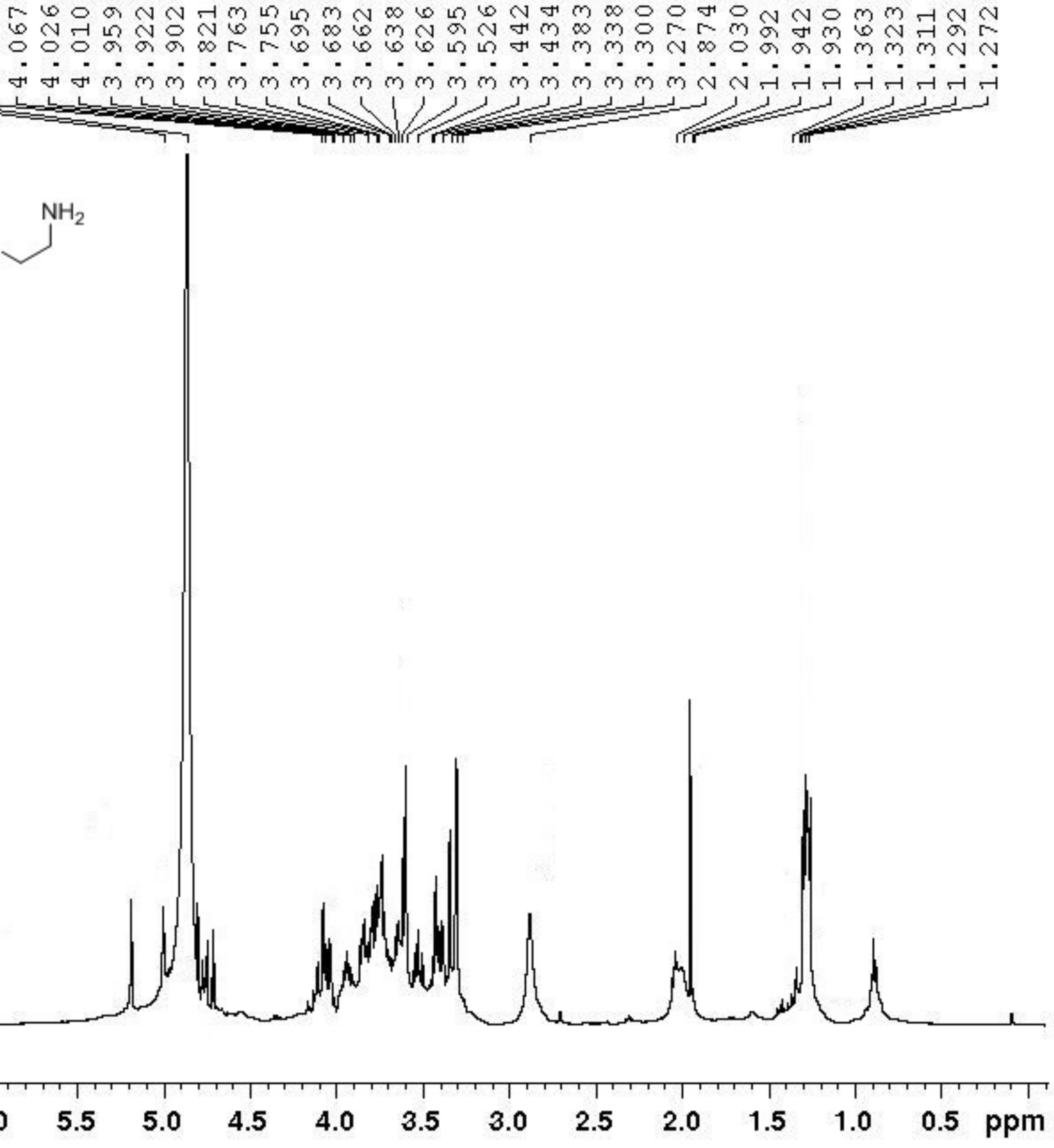

BRUKER

Current Data Parameters	
NAME	BM-DP-090
EXPNO	80
PROCNO	1
F2 - Acquisition Parameters	
Date_	20181126
Time	15.32
INSTRUM	Bruker_default_av500
PROBHD	5 mm PABBO BB/
PULPROG	zg30
TD	66560
SOLVENT	MeOD
NS	32
DS	2
SWH	10000.000 Hz
FIDRES	0.150240 Hz
AQ	3.3280001 sec
RG	50.8
DW	50.000 usec
DE	6.50 usec
TE	280.0 K
D1	1.00000000 sec
TD0	1
-------- CHANNEL f1 --------	
SFO1	500.3430898 MHz
NUC1	1H
P1	17.50 usec
PLW1	10.00000000 W
F2 - Processing parameters	
SI	131072
SF	500.3400147 MHz
WDW	EM
SSB	0
LB	0.30 Hz
GB	0
PC	1.00

GROUP BM
BM-DP-090 in CD3OD

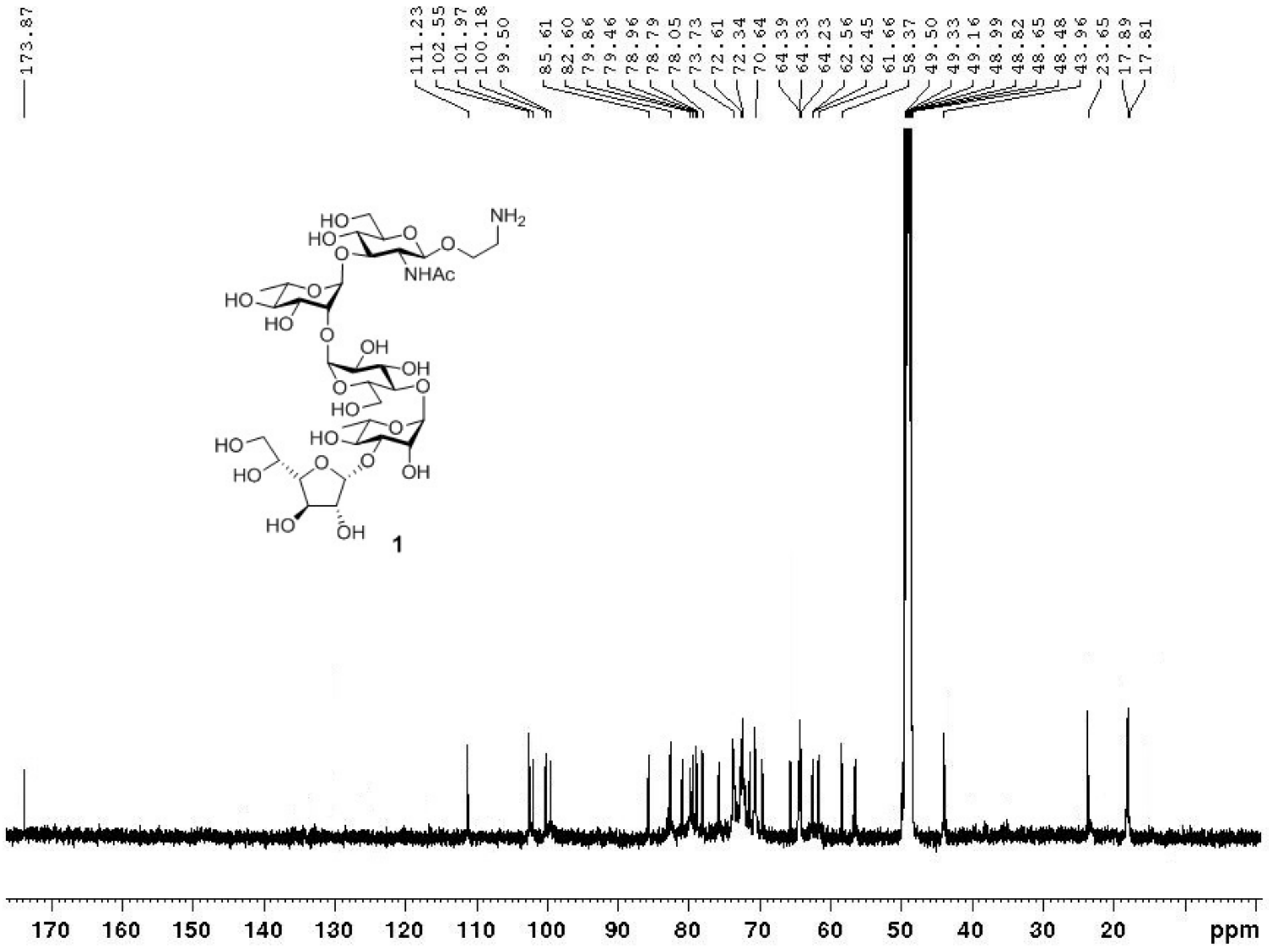

Supplement: File 2 — Copies of the 1H and 13C NMR spectra of all new compounds. [file Beilstein_J_Org_Chem-15-2563-s002.pdf]
